# Trends in antimicrobial resistance amongst pathogens isolated from blood and cerebrospinal fluid cultures in Pakistan (2011-2015): A retrospective cross-sectional study

**DOI:** 10.1371/journal.pone.0250226

**Published:** 2021-04-26

**Authors:** Nida Javaid, Qamar Sultana, Karam Rasool, Sumanth Gandra, Fayyaz Ahmad, Safee Ullah Chaudhary, Shaper Mirza

**Affiliations:** 1 Department of Biology, School of Science and Engineering, Lahore University of Management Science, Lahore, Pakistan; 2 Department of Microbiology, Chughtai Lab/Chughtai Institute of Pathology, Lahore, Pakistan; 3 Division of Infectious Diseases, Washington University School of Medicine in St. Louis, St. Louis, MI, United States of America; 4 Department of Statistics, University of Gujrat, Gujrat, Pakistan; 5 Biomedical Informatics Research Laboratory, Department of Biology, Lahore University of Management Sciences, Lahore, Pakistan; Nitte University, INDIA

## Abstract

While antimicrobial resistance (AMR) continues to be a major public health problem in Pakistan, data regarding trends of resistance among pathogenic bacteria remains scarce, with few studies presenting long-term trends in AMR. This study was therefore designed to analyze long-term AMR trends at a national level in Pakistan. We report here results of a comprehensive analysis of resistance, among pathogens isolated from blood and cerebrospinal fluid (CSF), between 2011 and 2015. Susceptibility data was obtained from a local laboratory with collection points all across Pakistan (Chughtai Laboratory). Resistance proportions to most commonly used antimicrobials were calculated for each pathogen over a period of five years. While *Acinetobacter* species demonstrated highest resistance rates to all tested antimicrobials, a sharp increase in carbapenem resistance was the most noticeable (50%-95%) between 2011–2015. Our results also highlight the presence of third and fourth generation cephalosporins resistance in *Salmonella enterica* serovar Typhi in Pakistan. Interestingly, where rise in AMR was being observed in some major invasive pathogens, decreasing resistance trends were observed in *Staphylococcus aureus*, against commonly used antimicrobials. Overall pathogens isolated from blood and CSF between 2011–2015, showed an increase in resistance towards commonly used antimicrobials.

## Introduction

The use of broad-spectrum antimicrobials for treatment of invasive infections, defined as bloodstream and cerebrospinal fluid (CSF) infections, has resulted in an increase in antimicrobial resistance (AMR). As a consequence, the treatment of such infections is becoming increasingly difficult leading to treatment failures and increased mortality. Availability of over the counter drugs in developing countries such as Pakistan has led to the epidemic of AMR in these countries. Indiscriminate usage of antimicrobials exerts an increased selection pressure on the bacterial population resulting in accelerated emergence of AMR [1]. Recent estimates suggested that invasive infections, in particular antimicrobial resistant invasive infections, account for 5.3 million deaths around the world annually. A significant proportion of these deaths occurs in low to middle income countries (LMIC) such as Pakistan [2]. While developing countries are battling an accelerated spread of AMR, developed countries are also experiencing the same trend. In the United States between 1999 and 2012, 47.9% *Acinetobacter baumanii* were carbapenem resistant, 68.4% *Staphylococcus epidermidis* were ciprofloxacin resistant, and 13.7% *Escherichia coli* (*E*. *coli*) were β-lactam resistant [3–5]. Furthermore, a similar resistance landscape had emerged in Canada between 2007 and 2011, where 27% of *E*.*coli* were resistant to ciprofloxacin, 19.3% *Staphylococcus aureus (S*. *aureus*) were resistant to methicillin, and 16.8% *Streptococcus pneumoniae* (*S*. *pneumoniae*) were resistant to penicillin [6].

Unavailability of reliable data in the developing countries like Pakistan, makes it difficult to develop efficient methods to monitor and control AMR [1]. The limited number of studies undertaken to investigate resistance in Pakistan indicate that most of the pathogens are resistant to commonly used antibiotics. For instance, between 1997 and 2014, 91% of *E*. *coli* were reported to be resistant to amikacin, while 91.7% *Salmonella enterica* serovar Typhi (*S*. Typhi) were fluoroquinolone resistant, *and* 90.9% *Acinetobacter* species were imipenem resistant [7–9]. These studies indicate that AMR poses a burgeoning public health problem in Pakistan and highlights the need of an in-depth assessment of AMR situation.

Towards this goal, the present study examined AMR data from a large diagnostic lab to identify (i) pathogens that are most commonly isolated from blood and CSF cultures in Pakistan, (ii) the patterns of resistance amongst these pathogens, and (iii) their co-resistance trends.

## Materials and methods

For this retrospective cross-sectional study, AMR data on pathogens isolated between 2011–2015 and their antimicrobial susceptibilities were obtained from Chughtai Lab (CL), a local diagnostic facility with over 180 collection centers in 60 cities of Pakistan. Standard biochemical tests and Analytical Profile Index (API) identification kits (BioMerieux) [10] were used to identify bacterial species at CL. Antimicrobial susceptibility testing was done using disc diffusion method following Clinical Laboratory & Standards Institute (CLSI) guidelines [11].

Data on positive cultures were obtained from an electronic database containing patients’ reports. Information obtained on each case included: (1) patient’s age, (2) patient’s sex, (3) year of sample collection, (4) specimen source, (5) city in which the sample was drawn, (6) isolated pathogen in the positive culture, and (7) susceptibility results (defined as susceptible, intermediate, or resistant).

**Fig 1:**
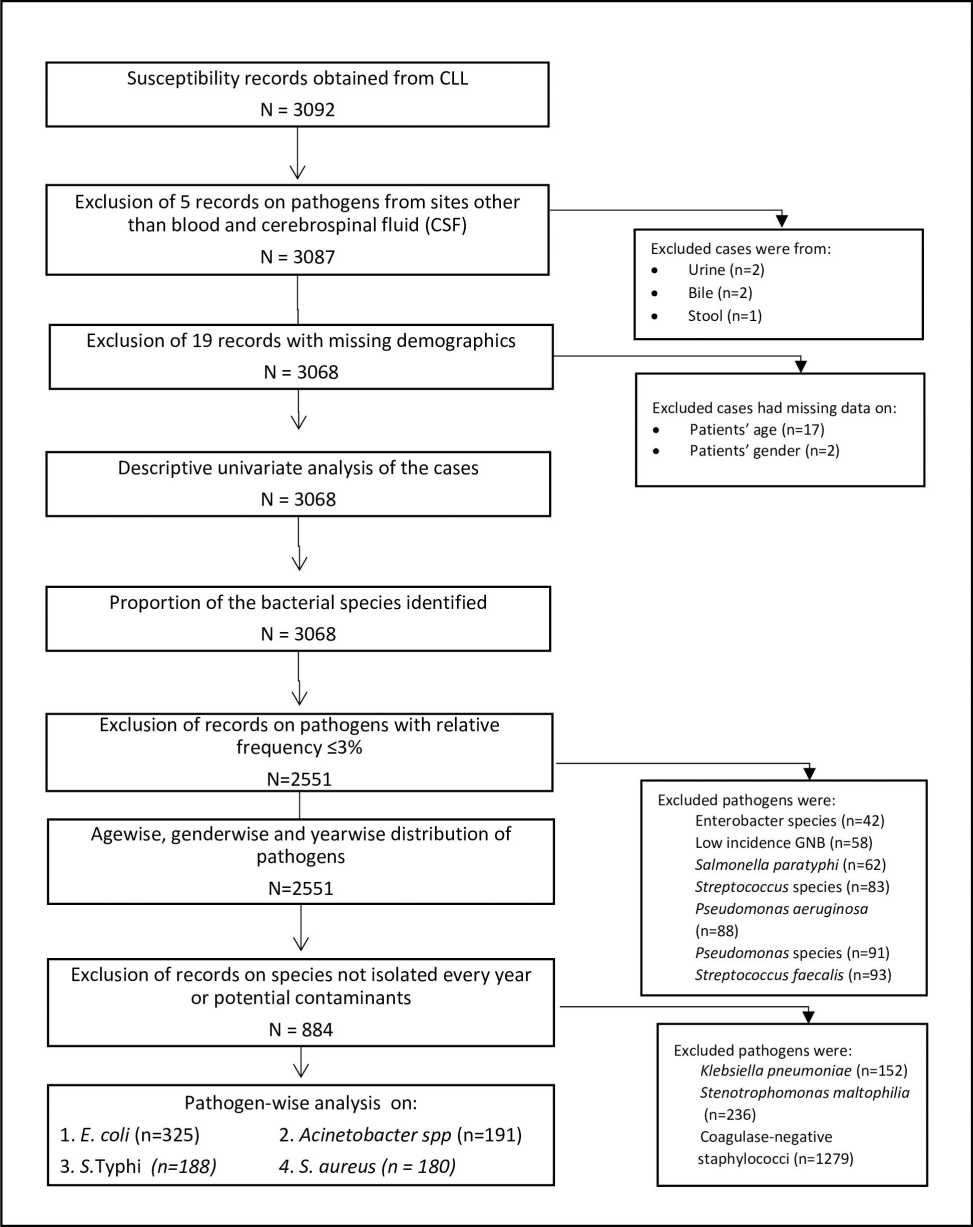
Flow of cases. Flow of the cases through the study has been shown.

Of a total of 3092 cases, datasets with missing information on age (n = 17) and sex (n = 2) were excluded from the study. Cases with information on pathogens isolated from sites other than blood and CSF (n = 5) were also excluded from the dataset (Fig 1). Most commonly isolated microorganisms from blood and CSF cultures were identified and used for all later analysis. Microorganisms that demonstrated low frequency of isolation and were found in less than 100 samples were also eliminated from the analysis. Pathogens were stratified by year of sample collection and patients’ age (<5 years, 6–18 years, 19–45 years, 46–65 years, and 65< years), and patients’ sex. For each case, intermediate resistance was considered as resistant. Susceptibility for all tested antimicrobials was examined and reported in each species separately. The bacterial species analyzed included *E*. *coli*, *Acinetobacter* species, *S*. *aureus*, and *S*. Typhi.

### Statistical analysis

Statistical analyses were performed using IBM SPSS statistics 22 (SPSS Inc., Chicago, Ill., USA). SAS university edition software (Cary, NC: SAS Institute Inc) was employed to perform Cochran Armitage test for trends. Figures were plotted using Circa software (www.omgenomics.com). P-value of <0.05 was considered significant for all statistical analyses. Frequency of cases was calculated by pathogens, by age and sex of patients, site of infection, and year of isolation using univariate analysis. Unadjusted resistance rates in pathogens were determined over age groups, sex, and year of isolation using univariate analysis. As the number of isolates varied annually throughout the study period, proportions of the resistant pathogens were evaluated to normalize the data. The proportions of resistant isolates were examined over age and sex respectively using Fisher’s exact test, with Bonferroni corrections. Cochran Armitage test for trends was used to investigate trends of AMR over time. Pairwise resistance (referred to as co-resistance) trends were examined using chi-square test of independence. For significantly associated pairwise resistance, binary logistic regression was used to calculate odds ratios, which outlined odd of resistance to one antimicrobial given resistance to another antimicrobial. Antimicrobials with an overall resistance above 1.5% were plotted in a Circos-based AMR map for each pathogen.

## Results

### Geographical, temporal, and demographical distribution of participants

Burden of invasive infections vary between different geographic regions as well as patients’ demographics [2, 12]. Hence, we wanted to profile the distribution of bacterial pathogens isolated from blood and CSF cultures over patients’ demographics, geographic location and year of isolation. Results of univariate analysis of demographic distribution of pathogens associated with invasive infections and the phenotypic characteristics of such pathogens, are presented in Table 1.

**Table 1:** Attributes of pathogens isolated from blood and cerebrospinal fluid between 2011–2015 in Pakistan.

	Number of bacterial pathogens (n = 3068)
**Age** (years)	
≤5	1061 (34.6%)
6–18	262 (8.5%)
19–45	711 (23.2%)
46–65	550 (17.9%)
>65	484 (15.8%)
**Sex**	
Female	1245 (40.6%)
Male	1823 (59.4%)
**Site of Infection**	
Blood	2917 (95.1%)
CSF	151 (4.9%)
**Year of Isolation**	
2011	262 (8.5%)
2012	296 (9.6%)
2013	493 (16.1%)
2014	750 (24.4%)
2015	1267 (41.3%)
**Location of sample collection**	
Punjab	2701 (87.9%)
KPK	338 (11%)
Sindh	25 (1%)
Baluchistan	1 (0%)
No information	3 (.1%)
**Phenotypic characters/Gram Staining**	
Gram-positive bacteria	1433 (46.7%)
Gram-negative bacteria	1635 (53.3%)

A total of 3068 microorganisms were isolated from blood and CSF cultures between 2011 and 2015. These cases were reported from 44 cities, from all four provinces of Pakistan, however, majority of cases were reported from 13 cities, whereas the remaining 31 cities reported less than 11 cases. The median number of cases from any given city was 4 with an interquartile range of 2–15. Analysis by province indicated that highest number of cases were reported from Punjab (87.9%), followed by Khyber Pakhtunkhwa (11%), Sindh (1%) and Baluchistan (0.033%). Within Punjab most (60.5%) of reported cases were from the city of Lahore, which is the most populous city of Punjab followed by Faisalabad (18.3%) and Abbottabad (8.3%). Detailed distribution of cases over geographic location is given in the S1 Table.

### Temporal and demographical distribution of pathogens frequently isolated from blood and CSF cultures

Distribution of isolates by patient characteristics (age and sex) and by year is given in Table 2. Previous studies have shown that the incidence rates of pathogen-specific invasive infections fluctuate on the basis of their temporal, spatial, and demographical characteristics [13]. Hence, the next logical step was to determine the distribution patterns of pathogens isolated from blood and CSF specimens. To investigate the temporal and demographical distribution of the bacterial species, univariate analyses were performed. Over 75% of these pathogens were from one of the seven most common bacterial species. These included CoNS (41.7%), *E*. *coli* (10.6%), *Stenotrophomonas*. *maltophilia* (*S*. *maltophilia*) *(*6.2%), *Acinetobacter* species (6.2%), *S*. Typhi (6.1%), *S*. *aureus* (5.9%), and *Klebseilla pneumoniae* (*K*. *pneumoniae*) (5%). Since data was collected from a diagnostic laboratory, it was difficult to obtain data on patient characteristics such as clinical manifestation as well as mortality and morbidity rates for CoNS. Therefore, samples containing CoNS were eliminated from the study (please see discussion for further information on CoNS in Pakistan).

**Table 2:** Profile of common bacterial species isolated from blood and cerebrospinal fluid (CSF) cultures with corresponding demographical and temporal distribution.

Organism	Age (years)					Sex		Year					Total
	<5	6–18	19–45	46–65	>65	Female	Male	2011	2012	2013	2014	2015	
Coagulase-negative staphylococci	444 (41.8%)	74 (28.2%)	297 (41.8%)	255 (19.9%)	209 (43.2%)	557 (44.7%)	722 (39.6%)	101 (38.5%)	111 (37.5%)	198 (40.2%)	337 (44.9%)	532 (42%)	1279 (41.7%)
*Escherichia coli*	57 (5.4%)	11 (4.2%)	58 (8.2%)	90 (27.7%)	109 (22.5%)	144 (11.6%)	181 (9.9%)	44 (16.8%)	53 (17.9%)	73 (14.8%)	66 (8.8%)	89 (7%)	325 (10.6%)
*Stenotrophomonas maltophilia*	215 (20.3%)	3(1.15%)	8 (1.1%)	3(0.55%)	7 (1.4%)	76 (6.1%)	160 (8.8%)	0	0	5(1.01%)	46 (6.1%)	185 (14.6%)	236 (7.7%)
*Acinetobacter* species	85 (8%)	9 (3.4%)	41 (5.8%)	35 (18.3%)	21 (4.3%)	81 (6.5%)	110 (6%)	15 (5.7%)	16 (5.4%)	26 (5.3%)	44 (5.9%)	90 (7.1%)	191 (6.2%)
*Salmonella enterica* serovar Typhi	16 (1.5%)	82 (31.3%)	86 (12.1%)	3(0.55%)	1(0.21%)	81 (6.5%)	107 (5.9%)	13 (5%)	20 (6.8%)	33 (6.7%)	58 (7.7%)	64 (5.1%)	188 (6.1%)
*Staphylococcus aureus*	41 (3.9%)	13 (5%)	52 (7.3%)	45 (25%)	29 (6%)	71 (5.7%)	109 (6%)	19 (7.3%)	20 (6.8%)	33 (6.7%)	47 (6.3%)	61 (4.8%)	180 (5.9%)
*Klebsiella pneumoniae*	66 (6.2%)	8 (3.1%)	26 (3.7%)	29 (19.1%)	23 (4.8%)	53 (4.3%)	99 (5.4%)	1(0.38%)	0	29 (5.9%)	51 (6.8%)	71 (5.6%)	152 (5%)

Data are n (% isolates in a column). Bacterial species with relative frequency above 5% are shown.in the table.

#### Antimicrobial resistance (AMR) trends in pathogens isolated from blood and CSF cultures

**Fig 2:**
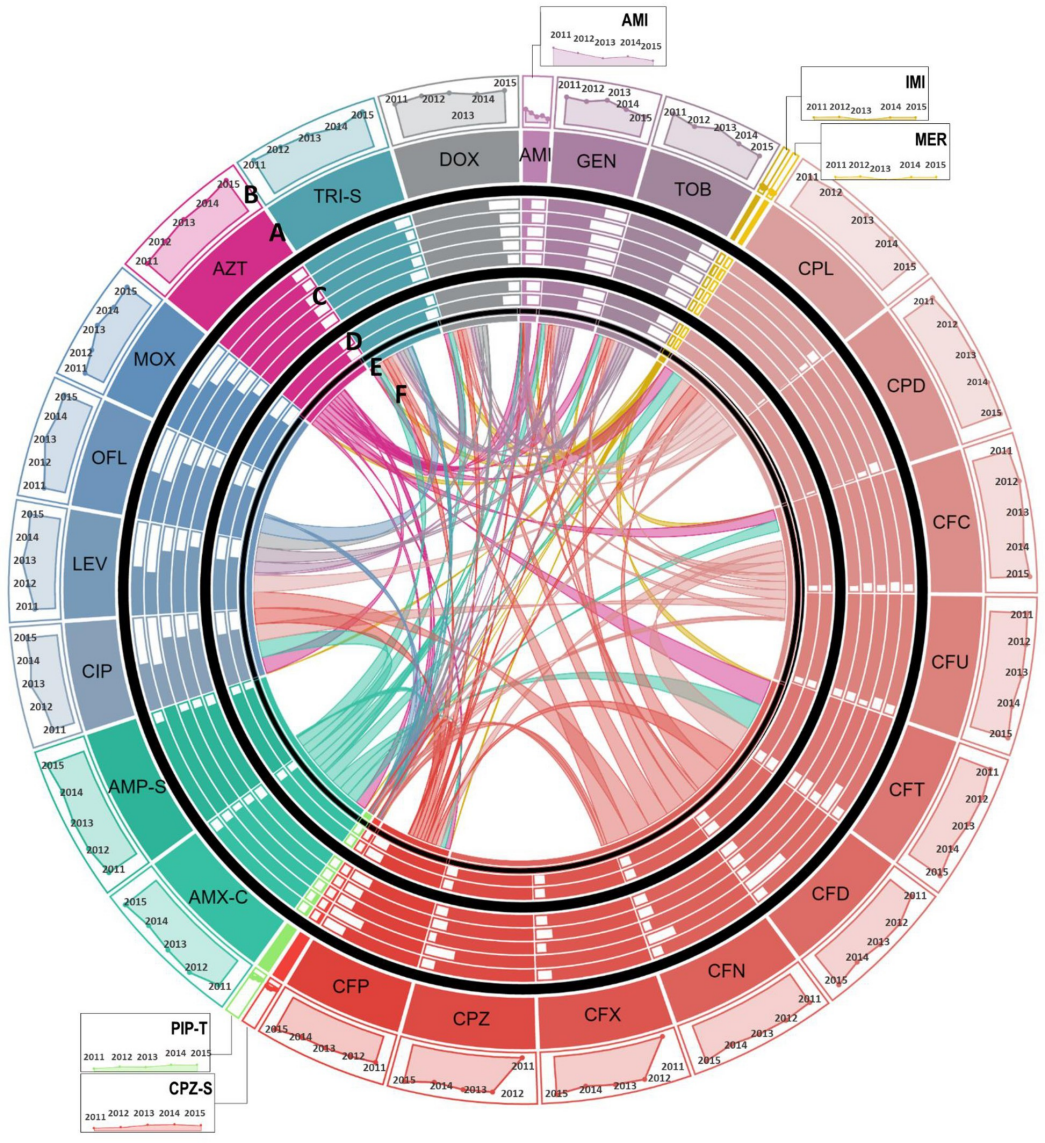
Antimicrobial resistance in *Escherichia coli* (*E*. *coli*). **A:** Each section of the diagram represents the resistance observed in *E*. *coli* against the antibiotic. Size of each section is proportional to the proportion of *E*. *coli* resistant to the antibiotic over the study period. Antibiotics of the same class are shown in similar colors. **B:** Line graphs show temporal trends of proportion of resistant *E*. *coli* in a clockwise direction from 2011 to 2015. **C:** Bar charts show the comparison of susceptibility to resistant strains in patients of different age groups. Moving from out to inward, bars represent proportion of resistant *E*. *coli* reported in children <5 years of age, young adults between 6 to 18 years, middle aged 19 to 45 years old, 45 to 65 years old patients, and elderly over 65 years of age, respectively. **D:** Gender-wise comparison to susceptibility to resistant *E*. *coli* is shown in form bars. Outer circle and inner circle shows proportion of resistant *E*. *coli* isolated from women vs. men, respectively. **E:** For co-resistance analysis, antibiotics belonging to the same class with same susceptibility profile for all isolates of *E*. *coli* were merged into a single variable. **F:** Proportion of *E*. *coli* isolates resistant to one antimicrobial resistant to another antimicrobial are shown in the connections. The area covered by the connection on E is proportional to the level of co-resistance observed. Co-resistance proportions were scaled down to 1/15^th^ of the actual overlap for visualization. **Abbreviations:** AMI: Amikacin, GEN: Gentamicin, TOB: Tobramycin, IMI: Imipenem, MER: Meropenem, CPL: Cephalexin, CPD: Cephradine, CFC: Cefaclor, CFU: Cefuroxime, CFT: Cefotaxime, CFD: Ceftazidime, CFN: Ceftriaxone, CFX: Cefixime, CPZ: Cefoperazone, CFP: Cefepime, CPZ-S: Cefoperazone-Sulbactam, PIP-T: Piperacillin-Tazobactam, AMX-C: Amoxicillin-Clavulanic acid, AMP-S: Ampicillin-Sulbactam, CIP: Ciprofloxacin, LEV: Levofloxacin, OFL: Ofloxacin, MOX: Moxifloxacin, AZT: Aztreonam, TRI-S: Trime-Sulphamethoxazole, and DOX: Doxycycline.

**Fig 3:**
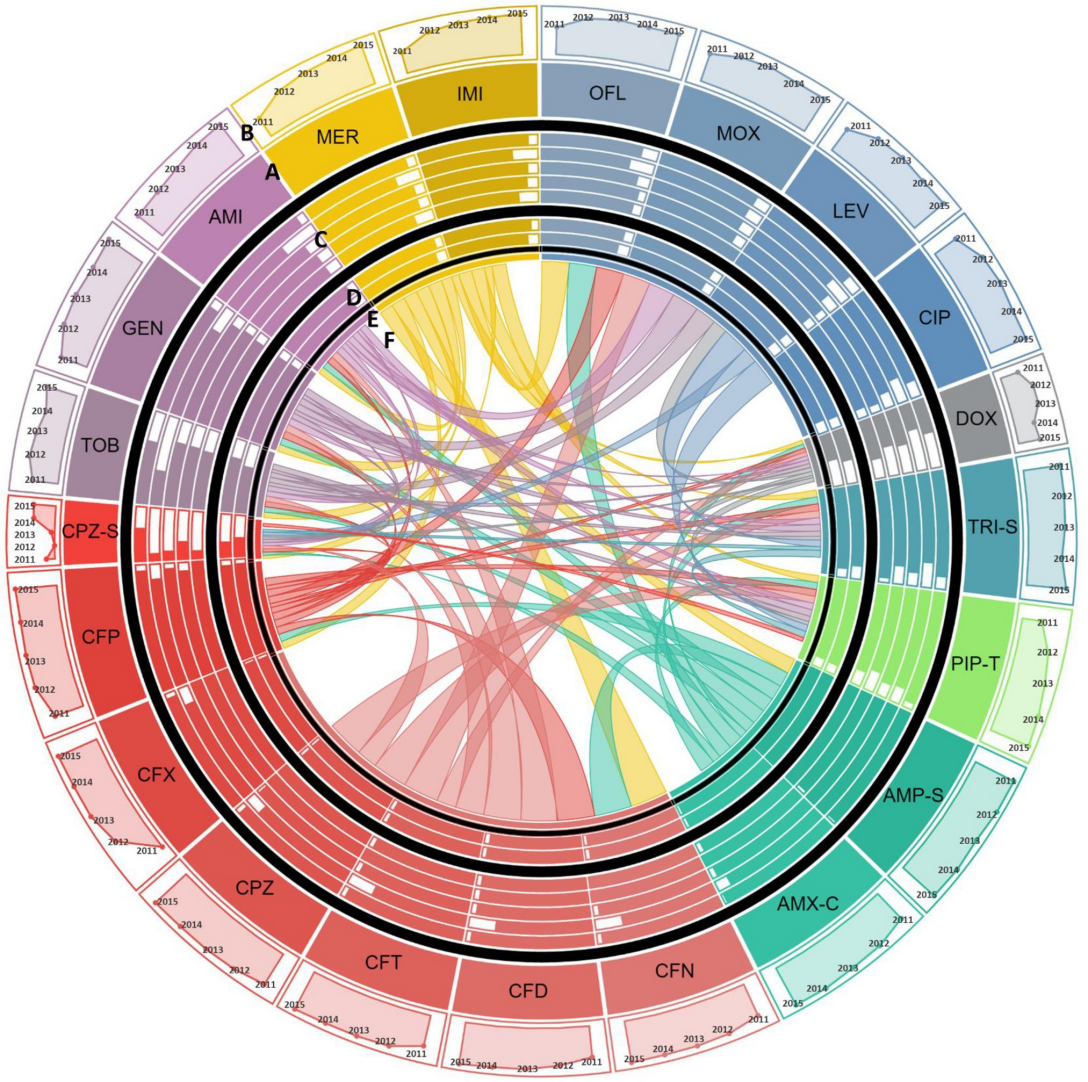
Antimicrobial resistance in *Acinetobacter*. **A:** Each section of the diagram represents the resistance observed in *Acinetobacter* species against the antibiotic. Size of each section is proportional to the proportion of *Acinetobacter* species resistant to the antibiotic over the study period. Antibiotics of the same class are shown in similar colors. **B:** Line graphs show temporal trends of proportion of resistant *Acinetobacter* species in a clockwise direction from 2011 to 2015. **C:** Bar charts show the comparison of susceptibility to resistant isolates in patients of different age groups. Moving from out to inward, bars represent proportion of resistant *Acinetobacter* species reported in children <5 years of age, young adults between 6 to 18 years, middle aged 19 to 45 years old, 45 to 65 years old patients, and elderly over 65 years of age, respectively. **D:** Gender-wise comparison to susceptibility to resistant *Acinetobacter* species is shown in form bars. Outer circle and inner circle shows proportion of resistant *Acinetobacter* species isolated from women vs. men, respectively. **E:** For co-resistance analysis, antibiotics belonging to the same class with same susceptibility profile for all isolates of *E*. *coli* were merged into a single variable. **F:** Proportion *Acinetobacter* species isolates resistant to one antimicrobial resistant to another antimicrobial are shown in the connections. The area covered by the connection on E is proportional to the level of co-resistance observed. Co-resistance proportions were scaled down to 1/10^th^ of the actual overlap for visualization. **Abbreviations:** DOX: Doxycycline, TRI-S: Trime-Sulphamethoxazole, PIP-T: Piperacillin-Tazobactam, AMP-S: Ampicillin-Sulbactam, AMX-C: Amoxicillin-Clavulanic acid, CIP: Ciprofloxacin, LEV: Levofloxacin, OFL: Ofloxacin, MOX: Moxifloxacin, CFN: Ceftriaxone, CFD: Ceftazidime, CFT: Cefotaxime, CPZ: Cefoperazone, CFX: Cefixime, CFP: Cefepime, CPZ-S: Cefoperazone-Sulbactam, IMI: Imipenem, MER: Meropenem, TOB: Tobramycin, GEN: Gentamicin, and AMI: Amikacin.

**Fig 4:**
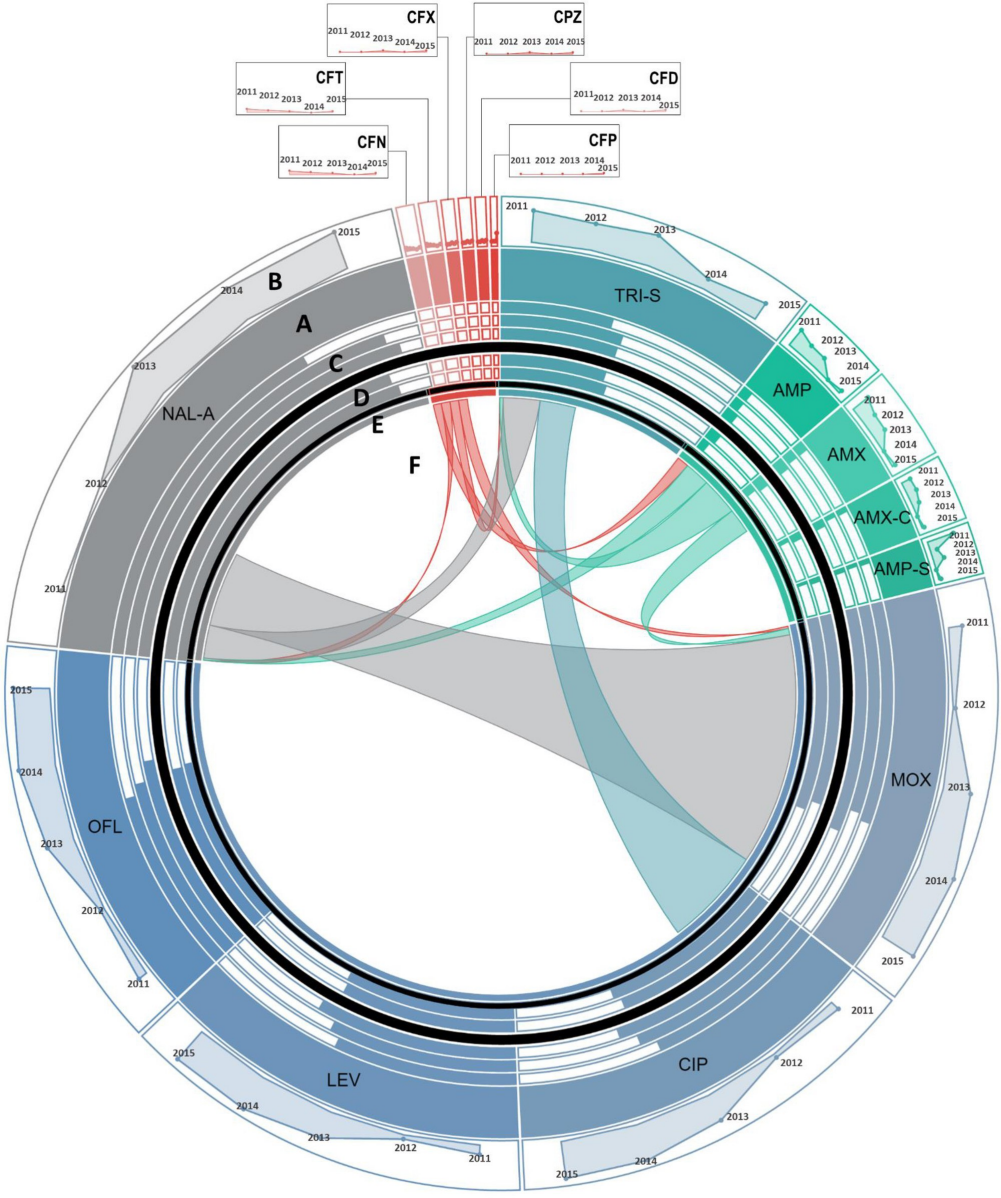
Antimicrobial resistance in *Salmonella enterica* serovar Typhi *(S*. Typhi*)*. **A:** Each section of the diagram represents the resistance observed in *S*. Typhi against the antibiotic. Size of each section is proportional to the proportion of *S*. Typhi resistant to the antibiotic over the study period. Antibiotics of the same class are shown in similar colors. **B:** Line graphs show temporal trends of proportion of resistant *S*. Typhi in a clockwise direction from 2011 to 2015. **C:** Bar charts show the comparison of susceptibility to resistant isolates in patients of different age groups. Moving from out to inward, bars represent proportion of resistant *S*. Typhi reported in children <5 years of age, young adults between 6 to 18 years, and middle aged 19 to 45 years old, respectively. **D:** Gender-wise comparison to susceptibility to resistant *S*. Typhi is shown in form bars. Outer circle and inner circle show proportion of resistant *S*. Typhi isolated from women vs. men, respectively. **E:** For co-resistance analysis, antibiotics belonging to the same class with same susceptibility profile for all isolates of *E*. *coli* were merged into a single variable. **F:** Proportion *S*. Typhi isolates resistant to one antimicrobial resistant to another antimicrobial are shown in the connections. The area covered by the connection on E is proportional to the level of co-resistance observed. Co-resistance proportions were scaled down to 1/10^th^ of the actual overlap for visualization. **Abbreviations:** CFN: Ceftriaxone, CFD: Ceftazidime, CFT: Cefotaxime, CPZ: Cefoperazone, CFX: Cefixime, CFP: Cefepime, CIP: Ciprofloxacin, LEV: Levofloxacin, OFL: Ofloxacin, MOX: Moxifloxacin, NAL-A: Nalidixic acid, AMP-S: Ampicillin-Sulbactam, AMX-C: Amoxicillin-Clavulanic acid, AMP: Ampicillin, AMX: Amoxicillin, and TRI-S: Trime-Sulphamethoxazole.

**Fig 5:**
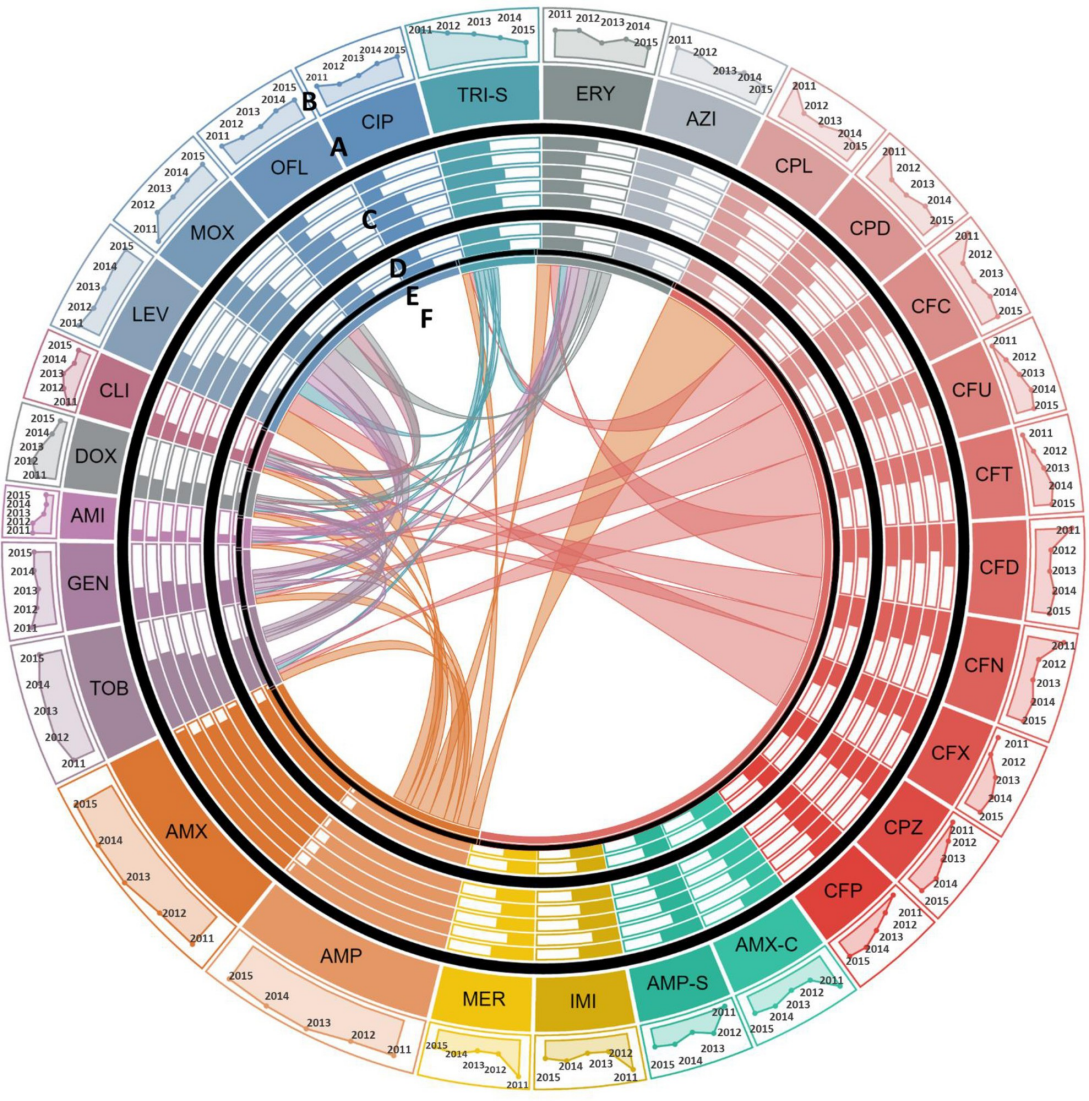
Antimicrobial resistance in *Staphylococcus aureus (S*. *aureus*). **A:** Each section of the diagram represents the resistance observed in *S*. *aureus* against the antibiotic. Size of each section is proportional to the proportion of *S*. *aureus* resistant to the antibiotic over the study period. Antibiotics of the same class are shown in similar colors. **B:** Line graphs show temporal trends of proportion of resistant *S*. *aureus* in a clockwise direction from 2011 to 2015. **C:** Bar charts show the comparison of susceptibility to resistant strains in patients of different age groups. Moving from out to inward, bars represent proportion of resistant *S*. *aureus* reported children in <5 years of age, young adults between 6 to 18 years, middle aged 19 to 45 years old, 45 to 65 years old patients, and elderly over 65 years of age, respectively. **D:** Gender-wise comparison to susceptibility to resistant *S*. *aureus* is shown in form bars. Outer circle and inner circle show proportion of resistant *S*. *aureus* isolated from women vs. men, respectively. **E:** For co-resistance analysis, antibiotics belonging to the same class with same susceptibility profile for all isolates of *S*. *aureus* were merged into a single variable. **F:** Proportion of *S*. *aureus* isolates resistant to one antimicrobial resistant to another antimicrobial are shown in the connections. The area covered by the connection on E is proportional to the level of co-resistance observed. Co-resistance proportions were scaled down to 1/10^th^ of the actual overlap for visualization. **Abbreviations:** AMI: Amikacin, GEN: Gentamicin, TOB: Tobramycin, AMP: Ampicillin, AMX: Amoxicillin, IMI: Imipenem, MER: Meropenem, CPL: Cephalexin, CPD: Cephradine, CFC: Cefaclor, CFU: Cefuroxime, CFT: Cefotaxime, CFD: Ceftazidime, CFN: Ceftriaxone, CFX: Cefixime, CPZ: Cefoperazone, CFP: Cefepime, AMX-C: Amoxicillin-Clavulanic acid, AMP-S: Ampicillin-Sulbactam, CIP: Ciprofloxacin, LEV: Levofloxacin, OFL: Ofloxacin, MOX: Moxifloxacin, CLI: Clindamycin, AZI: Azithromycin, ERY: Erythromycin, TRI-S: Trime-Sulphamethoxazole, and DOX: Doxycycline.

Variations in the treatment approaches of physicians within a geographical location, patients’ compliance, and the ability of pathogens to acquire and disseminate resistance, impact resistance trends in different pathogens [14]. The ability of resistant pathogens to cause infections is also dependent on host-related factors including age, gender, and co-morbidities [15]. Towards this, we determined temporal and demographical AMR trends in pathogens most frequently isolated from blood and CSF. Susceptibility data was not available for all years throughout the study period on *S*. *maltophilia* and *K*. *pneumoniae*, therefore these two pathogens were excluded from the analysis. Moreover, in the absence of clinical data on patients to support CoNS role in disease manifestation, CoNS was taken out of the downstream analysis. Each of the remaining four pathogens were analyzed separately. For each isolate, susceptibility data for all antimicrobials was not available. To account for missing values, available case approach was employed to analyze resistance trends for each antimicrobial. As a result, the number of data points (n) varied between analyses involving different antimicrobials in a pathogen. Tables 3–6 and Figs 2–5 demonstrate resistance trends in *E*. *coli*, *Acinetobacter* species, *S*. Typhi and *S*. *aureus*.

**Table 3:** Temporal, age-wise, and gender-wise prevalence of resistant *Escherichia coli* strains isolated from blood and cerebrospinal fluid (CSF) cultures in Pakistan (2011–2015).

Antimicrobials	Antimicrobial group	Prevalence (%) of resistant organisms isolated from patients of different age-groups						Prevalence (%) of resistant organisms isolated from males and female patients			Year-wise prevalence (%) of resistant organisms						Total
		<5 Years	6–18 Years	19–45 Years	46–65 Years	>65 Years	P for difference	Female	Male	P for difference	2011	2012	2013	2014	2015	P for trend	
**Cephalexin**	Cephalosporins	56/56 (100%)	10/11 (90.9%)	55/58 (94.8%)	88/90 (97.8%)	107/109 (98.2%)	NS	139/144 (96.5%)	177/180 (98.3%)	NS	37/43 (86%)	52/53 (98.1%)	72/73 (98.6%)	66/66 (100%)	89/89 (100%)	< .0001	316/324 (97.5%)
**Cephradine**	Cephalosporins	56/56 (100%)	10/11 (90.9%)	55/58 (94.8%)	88/90 (97.8%)	107/109 (98.2%)	NS	139/144 (96.5%)	177/180 (98.3%)	NS	37/43 (86%)	52/53 (98.1%)	72/73 (98.6%)	66/66 (100%)	89/89 (100%)	< .0001	316/324 (97.5%)
**Cefaclor**	Cephalosporins	53/57 (93%)	10/11 (90.9%)	54/58 (93.1%)	82/90 (91.1%)	102/109 (93.6%)	NS	133/144 (92.4%)	168/181 (92.8%)	NS	40/44 (90.9%)	52/53 (98.1%)	68/73 (93.2%)	56/66 (84.8%)	85/89 (95.5%)	NS	301/325 (92.6%)
**Cefuroxime**	Cephalosporins	52/57 (91.2%)	8/9 (88.9%)	51/54 (94.4%)	79/87 (90.8%)	96/105 (91.4%)	NS	124/136 (91.2%)	162/176 (92%)	NS	28/31 (90.3%)	49/53 (92.5%)	68/73 (93.2%)	56/66 (84.8%)	85/89 (95.5%)	NS	286/312 (91.7%)
**Ampicillin-Sulbactam**	Penicillin-Inhibitor	45/50 (90%)	7/7 (100%)	35/38 (92.1%)	61/67 (91%)	78/88 (88.6%)	NS	91/101 (90.1%)	135/149 (90.6%)	NS	17/21 (81%)	11/12 (91.7%)	57/62 (91.9%)	56/66 (84.8%)	85/89 (95.5%)	NS	226/250 (90.4%)
**Amoxicillin-Clavulanicacid**	Penicillin-Inhibitor	51/57 (89.5%)	10/11 (90.9%)	54/58 (93.1%)	82/90 (91.1%)	95/109 (87.2%)	NS	129/144 (89.6%)	163/181 (90.1%)	NS	33/44 (75%)	51/53 (96.2%)	67/73 (91.8%)	56/66 (84.8%)	85/89 (95.5%)	0.0284	292/325 (89.8%)
**Ceftriaxone**	Cephalosporins	51/57 (89.5%)	8/11 (72.7%)	53/58 (91.4%)	76/90 (84.4%)	93/109 (85.3%)	NS	125/144 (86.8%)	156/181 (86.2%)	NS	37/44 (84.1%)	47/53 (88.7%)	63/73 (86.3%)	52/66 (78.8%)	82/89 (92.1%)	NS	281/325 (86.5%)
**Cefixime**	Cephalosporins	38/44 (86.4%)	5/5 (100%)	35/39 (89.7%)	53/64 (82.8%)	73/84 (86.9%)	NS	81/93 (87.1%)	123/143 (86%)	NS		8/9 (88.9%)	62/72 (86.1%)	52/66 (78.8%)	82/89 (92.1%)	NS	204/236 (86.4%)
**Cefoperazone**	Cephalosporins	41/47 (87.2%)	5/7 (71.4%)	44/48 (91.7%)	62/74 (83.8%)	81/94 (86.2%)	NS	97/111 (87.4%)	136/159 (85.5%)	NS		36/42 (85.7%)	63/73 (86.3%)	52/66 (78.8%)	82/89 (92.1%)	NS	233/270 (86.3%)
**Trime-Sulphamethoxazole**	Sulphonamides	45/53 (84.9%)	10/11 (90.9%)	50/55 (90.9%)	76/86 (88.4%)	84/102 (82.4%)	NS	123/138 (89.1%)	142/169 (84%)	NS	42/44 (95.5%)	47/52 (90.4%)	58/67 (86.6%)	47/64 (73.4%)	71/80 (88.8%)	NS	265/307 (86.3%)
**Cefotaxime**	Cephalosporins	51/57 (89.5%)	8/11 (72.7%)	52/58 (89.7%)	75/89 (84.3%)	92/109 (84.4%)	NS	123/144 (85.4%)	155/180 (86.1%)	NS	35/44 (79.5%)	46/52 (88.5%)	63/73 (86.3%)	52/66 (78.8%)	82/89 (92.1%)	NS	278/324 (85.8%)
**Aztreonam**	Monobactams	45/52 (86.5%)	8/9 (88.9%)	35/42 (83.3%)	61/73 (83.6%)	77/94 (81.9%)	NS	98/117 (83.8%)	128/153 (83.7%)	NS	32/44 (72.7%)	13/17 (76.5%)	46/55 (83.6%)	53/65 (81.5%)	82/89 (92.1%)	0.0045	226/270 (83.7%)
**Ceftazidime**	Cephalosporins	49/56 (87.5%)	6/11 (54.5%)	51/58 (87.9%)	76/90 (84.4%)	89/109 (81.7%)	NS	119/144 (82.6%)	152/180 (84.4%)	NS	28/43 (65.1%)	46/53 (86.8%)	63/73 (86.3%)	52/66 (78.8%)	82/89 (92.1%)	0.0043	271/324 (83.6%)
**Doxycycline**	Tetracycline	36/51 (70.6%)	8/10 (80%)	43/54 (79.6%)	66/81 (81.5%)	91/106 (85.8%)	NS	114/134 (85.1%)	130/168 (77.4%)	NS	37/44 (84.1%)	47/53 (88.7%)	48/57 (84.2%)	48/66 (72.7%)	64/82 (78%)	NS	244/302 (80.8%)
**Cefepime**	Cephalosporins	44/57 (77.2%)	7/11 (63.6%)	47/58 (81%)	71/90 (78.9%)	82/109 (75.2%)	NS	111/144 (77.1%)	140/181 (77.3%)	NS	29/44 (65.9%)	38/53 (71.7%)	55/73 (75.3%)	50/66 (75.8%)	79/89 (88.8%)	0.0019	251/325 (77.2%)
**Moxifloxacin**	Fluoroquinolone	27/55 (49.1%)*	5/8 (62.5%)	40/54 (74.1%)	61/85 (71.8%)	84/104 (80.8%)#	0.001	93/134 (69.4%)	124/172 (72.1%)	NS	14/28 (50%)	31/50 (62%)	59/73 (80.8%)	47/66 (71.2%)	66/89 (74.2%)	0.0328	217/306 (70.9%)
**Ofloxacin**	Fluoroquinolone	29/57 (50.9%)*	5/11 (45.5%)	41/58 (70.7%)	66/90 (73.3%)	88/109 (80.7%)#	0.001	98/144 (68.1%)	131/181 (72.4%)	NS	23/44 (52.3%)	34/53 (64.2%)	59/73 (80.8%)	47/66 (71.2%)	66/89 (74.2%)	0.0198	229/325 (70.5%)
**Tobramycin**	Aminoglycosides	45/56 (80.4%)	8/11 (72.7%)	41/57 (71.9%)	59/90 (65.6%)	73/107 (68.2%)	NS	103/141 (73%)	123/180 (68.3%)	NS	33/43 (76.7%)	34/53 (64.2%)	59/73 (80.8%)	44/65 (67.7%)	56/87 (64.4%)	NS	226/321 (70.4%)
**Ciprofloxacin**	Fluoroquinolone	28/57 (49.1%)*	5/11 (45.5%)	43/58 (74.1%)	65/90 (72.2%)#	87/109 (79.8%)#	0.001	98/144 (68.1%)	130/181 (71.8%)	NS	24/44 (54.5%)	32/53 (60.4%)	59/73 (80.8%)	47/66 (71.2%)	66/89 (74.2%)	0.0188	228/325 (70.2%)
**Levofloxacin**	Fluoroquinolone	29/57 (50.9%)*	5/11 (45.5%)	41/58 (70.7%)	66/90 (73.3%)	87/109 (79.8%)#	0.001	97/144 (67.4%)	131/181 (72.4%)	NS	22/44 (50%)	34/53 (64.2%)	59/73 (80.8%)	47/66 (71.2%)	66/89 (74.2%)	0.0115	228/325 (70.2%)
**Gentamicin**	Aminoglycosides	40/57 (70.2%)	6/11 (54.5%)	34/58 (58.6%)	52/89 (58.4%)	63/109 (57.8%)	NS	91/144 (63.2%)	104/180 (57.8%)	NS	29/44 (65.9%)	32/53 (60.4%)	51/72 (70.8%)	38/66 (57.6%)	45/89 (50.6%)	0.0487 (-)	195/324 (60.2%)
**Amikacin**	Aminoglycosides	19/56 (33.9%)#	4/11 (36.4%)	10/57 (17.5%)	12/90 (13.3%)*	10/106 (9.4%)*	0.001	22/142 (15.5%)	33/178 (18.5%)	NS	14/44 (31.8%)	12/53 (22.6%)	10/73 (13.7%)	11/66 (16.7%)	8/84 (9.5%)	0.0018	55/320 (17.2%)
**Piperacillin-Tazobactam**	Penicillin-Inhibitor	10/55 (18.2%)#	3/11 (27.3%)#	5/56 (8.9%)	3/87 (3.4%)*	6/106 (5.7%)	0.005	8/140 (5.7%)	19/175 (10.9%)	NS	2/44 (4.5%)	4/53 (7.5%)	5/71 (7%)	7/63 (11.1%)	9/84 (10.7%)	NS	27/315 (8.6%)
**Cefoperazone-Sulbactam**	Cephalosporins-Inhibitor	13/57 (22.8%)#	2/11 (18.2%)	4/57 (7%)	2/87 (2.3%)*	6/108 (5.6%)*	0.001	8/141 (5.7%)	19/179 (10.6%)	NS	2/44 (4.5%)	3/53 (5.7%)	7/69 (10.1%)	7/65 (10.8%)	8/89 (9%)	NS	27/320 (8.4%)
**Imipenem**	Carbapenem	4/56 (7.1%)	3/11 (27.3%)#	3/58 (5.2%)	0/90 (0%)*	2/109 (1.8%)*	0.001	3/143 (2.1%)	9/181 (5%)	NS	2/43 (4.7%)	3/53 (5.7%)	0/73 (0%)	3/66 (4.5%)	4/89 (4.5%)	NS	12/324 (3.7%)
**Meropenem**	Carbapenem	4/56 (7.1%)	3/11 (27.3%)#	3/58 (5.2%)	0/90 (0%)*	2/109 (1.8%)*	0.001	3/143 (2.1%)	9/181 (5%)	NS	2/43 (4.7%)	3/53 (5.7%)	0/73 (0%)	3/66 (4.5%)	4/89 (4.5%)	NS	12/324 (3.7%)

Data are number of resistant isolates/total number of isolates (%). Denominator varies across cells because all isolates were not tested against the complete set of antimicrobials listed here. Empty cells indicate no cases were tested against the antimicrobial. P-value for difference was calculated by Fisher’s exact test. Bonferroni correction for pairwise comparisons was used to detect differences in percentage of resistant isolates between different age groups.

* indicates age groups less susceptible to resistant infections as compared to the age groups labeled with

#. P-value for trends was calculated by Cochran Armitage test for trends. Two-sided p-value has been reported. P for trend of <0.05 indicate an increasing resistance trend unless P-value is followed by “(-)” which shows a decreasing resistance rates against the antimicrobial in *Escherichia coli* strains. NS: Non significant p-value (i.e. >0.05).

**Table 4:** Temporal, age-wise and gender-wise prevalence of resistant Acinetobacter species strains isolated from blood and cerebrospinal fluid (CSF) cultures in Pakistan (2011–2015).

Antimicrobials	Antimicrobial group	Prevalence (%) of resistant organisms isolated from patients of different age-groups						Prevalence (%) of resistant organisms isolated from males and female patients			Year-wise prevalence (%) of resistant organisms						Total
		<5 Years	6–18 Years	19–45 Years	46–65 Years	>65 Years	P for difference	Female	Male	P for difference	2011	2012	2013	2014	2015	P for trends	
**Ampicillin-Sulbactam**	Penicillin-Inhibitor	79/81 (97.5%)	6/6 (100%)	29/29 (100%)	26/26 (100%)	15/15 (100%)	NS	59/59 (100%)	96/98 (98%)	NS	1/1 (100%)	4/4 (100%)	17/18 (94.4%)	44/44 (100%)	89/90 (98.9%)	NS	155/157 (98.7%)
**Cefixime**	Cephalosporins	79/83 (95.2%)	6/7 (85.7%)	32/32 (100%)	27/27 (100%)	15/15 (100%)	NS	66/67 (98.5%)	93/97 (95.9%)	NS		4/5 (80%)	24/25 (96%)	42/44 (95.4%)	89/90 (98.9%)	0.0476	159/164 (96.9%)
**Cefoperazone**	Cephalosporins	80/84 (95.2%)	6/7 (85.7%)	37/38 (97.4%)	33/33 (100%)	18/18 (100%)	NS	74/76 (97.4%)	100/104 (96.2%)	NS	5/6 (83.3%)	14/15 (93.3%)	24/25 (96%)	42/44 (95.4%)	89/90 (98.9%)	0.0397	174/180 (96.7%)
**Amoxicillin-Clavulanic acid**	Penicillin-Inhibitor	82/84 (97.6%)	8/9 (88.9%)	39/41 (95.1%)	35/35 (100%)	20/21 (95.2%)	NS	79/81 (97.5%)	105/109 (96.3%)	NS	12/15 (80%)	15/16 (93.7%)	24/25 (96%)	44/44 (100%)	89/90 (98.9%)	0.0006	184/190 (96.8%)
**Ceftriaxone**	Cephalosporins	81/85 (95.3%)	7/9 (77.8%)	38/40 (95%)	35/35 (100%)	20/20 (100%)	NS	78/81 (96.3%)	103/108 (95.4%)	NS	12/15 (80%)	14/15 (93.3%)	25/26 (96.1%)	41/43 (95.3%)	89/90 (98.9%)	0.003	181/189 (95.8%)
**Cefotaxime**	Cephalosporins	81/85 (95.3%)	7/9 (77.8%)	39/41 (95.1%)	35/35 (100%)	20/21 (95.2%)	NS	78/81 (96.3%)	104/110 (94.5%)	NS	11/15 (73.3%)	15/16 (93.7%)	25/26 (96.1%)	42/44 (95.4%)	89/90 (98.9%)	0.0004	182/191 (95.3%)
**Ceftazidime**	Cephalosporins	81/85 (95.3%)	7/9 (77.8%)	39/41 (95.1%)	33/34 (97.1%)	20/21 (95.2%)	NS	77/80 (96.2%)	103/110 (93.6%)	NS	11/15 (73.3%)	14/16 (87.5%)	24/25 (96%)	42/44 (95.4%)	89/90 (98.9%)	0.0001	180/190 (94.7%)
**Cefepime**	Cephalosporins	79/84 (94.0%)	7/8 (87.5%)	39/41 (95.1%)	33/35 (94.3%)	18/18 (100%)	NS	76/80 (95%)	100/106 (94.3%)	NS	7/10 (70%)	15/16 (93.7%)	25/26 (96.1%)	41/44 (93.2%)	88/90 (97.8%)	0.0064	176/186 (94.6%)
**Gentamicin**	Aminoglycosides	76/84 (90.5%)	7/9 (77.8%)	35/39 (89.7%)	32/34 (94.1%)	19/21 (90.5%)	NS	71/78 (91%)	98/109 (89.9%)	NS	9/14 (64.3%)	14/16 (87.5%)	24/26 (92.3%)	39/44 (88.6%)	83/87 (95.4%)	0.0021	169/187 (90.4%)
**Piperacillin-Tazobactam**	Penicillin-Inhibitor	73/78 (93.6%)	6/7 (85.7%)	33/38 (86.8%)	27/32 (84.4%)	17/20 (85%)	NS	66/73 (90.4%)	90/102 (88.2%)	NS	6/13 (46.1%)	12/16 (75%)	22/25 (88%)	34/37 (91.9%)	82/84 (97.6%)	< .0001	156/175 (89.1%)
**Imipenem**	Carbapenem	76/84 (90.5%)	7/9 (77.8%)	37/41 (90.2%)	31/34 (91.2%)	17/21 (80.9%)	NS	74/80 (92.5%)	94/109 (86.2%)	NS	7/14 (50%)	13/16 (81.2%)	23/26 (88.5%)	40/44 (90.9%)	85/89 (95.5%)	< .0001	168/189 (88.9%)
**Meropenem**	Carbapenem	76/84 (90.5%)	7/9 (77.8%)	36/40 (90%)	31/34 (91.2%)	17/21 (80.9%)	NS	73/79 (92.4%)	94/109 (86.2%)	NS	7/14 (50%)	12/15 (80%)	23/26 (88.5%)	40/44 (90.9%)	85/89 (95.5%)	< .0001	167/188 (88.8%)
**Moxifloxacin**	Fluoroquinolone	72/84 (85.7%)	6/7 (85.7%)	32/37 (86.5%)	28/31 (90.3%)	19/19 (100%)	NS	67/75 (89.3%)	90/103 (87.4%)	NS	4/6 (66.7%)	11/13 (84.6%)	23/25 (92%)	39/44 (88.6%)	80/90 (88.9%)	NS	157/178 (88.2%)
**Ofloxacin**	Fluoroquinolone	72/85 (84.7%)	7/9 (77.8%)	35/40 (87.5%)	33/35 (94.3%)	19/21 (90.5%)	NS	72/80 (90%)	94/110 (85.4%)	NS	9/14 (64.3%)	14/16 (87.5%)	24/26 (92.3%)	39/44 (88.6%)	80/90 (88.9%)	NS	166/190 (87.4%)
**Trimethoprim-Sulphamethoxazole**	Sulphonamides	70/80 (87.5%)	7/9 (77.8%)	35/40 (87.5%)	26/31 (83.9%)	19/20 (95%)	NS	69/80 (86.3%)	88/100 (88%)	NS	13/15 (86.7%)	14/16 (87.5%)	18/21 (85.7%)	33/43 (76.7%)	79/85 (92.9%)	NS	157/180 (87.2%)
**Amikacin**	Aminoglycosides	74/81 (91.4%)	6/9 (66.7%)	32/39 (82%)	29/33 (87.9%)	18/21 (85.7%)	NS	72/79 (91.1%)	87/104 (83.6%)	NS	11/15 (73.3%)	12/16 (75%)	21/26 (80.8%)	39/44 (88.6%)	76/82 (92.7%)	0.0058	159/183 (86.9%)
**Ciprofloxacin**	Fluoroquinolone	72/85 (84.7%)	7/9 (77.8%)	35/41 (85.4%)	33/35 (94.3%)	19/21 (90.5%)	NS	72/81 (88.9%)	94/110 (85.4%)	NS	9/15 (60%)	14/16 (87.5%)	24/26 (92.3%)	39/44 (88.6%)	80/90 (88.9%)	0.0396	166/191 (86.9%)
**Levofloxacin**	Fluoroquinolone	72/85 (84.7%)	7/9 (77.8%)	35/41 (85.4%)	33/35 (94.3%)	19/21 (90.5%)	NS	72/81 (88.9%)	94/110 (85.4%)	NS	9/15 (60%)	14/16 (87.5%)	24/26 (92.3%)	39/44 (88.6%)	80/90 (88.9%)	0.0396	166/191 (86.9%)
**Tobramycin**	Aminoglycosides	51/79 (64.6%)	3/8 (37.5%)	30/40 (75%)	26/34 (76.5%)	15/21 (71.4%)	NS	57/75 (76%)	68/107 (63.5%)	NS	9/15 (60%)	12/16 (75%)	18/26 (69.2%)	23/42 (54.8%)	63/83 (75.9%)	NS	125/182 (68.7%)
**Doxycycline**	Tetracycline	37/80 (46.2%)	3/8 (37.5%)	20/35 (57.1%)	16/31 (51.6%)	14/21 (66.7%)	NS	43/72 (59.7%)	47/103 (45.6%)	NS	6/14 (42.9%)	10/15 (66.7%)	11/17 (64.7%)	18/43 (41.9%)	45/86 (52.3%)	NS	90/175 (51.4%)
**Cefoperazone-Sulbactam**	Cephalosporins-Inhibitor	48/84 (57.1%)#	1/9 (11.1%)*	7/41 (17.1%)*	12/34 (35.3%)	3/20 (15%)*	< .0001	26/81 (32.1%)	45/107 (42.1%)	NS	3/15 (20%)	0/16 (0%)	1/25 (4%)	22/43 (51.2%)	45/89 (50.6%)	< .0001	71/188 (37.8%)

Data are number of resistant isolates/total number of isolates (%). Denominator varies across cells because all isolates were not tested against the complete set of antimicrobials listed here Empty cells indicate no cases were tested against the antimicrobial. P-value for difference was calculated by Fisher’s exact test. Bonferroni correction for pairwise comparisons was used to detect differences in percentage of resistant isolates between different age groups.

* indicates age groups less susceptible to resistant strains as compared to the age-groups labeled with

#. P-value for trends was calculated by Cochran Armitage test for trends. Two-sided p-value has been reported. P for trend of <0.05 indicate an increasing resistance trend. NS: Non significant p-value (i.e. >0.05)

**Table 5:** Temporal, age-wise and gender-wise prevalence of resistant *Salmonella enterica* serovar Typhi strains isolated from blood and cerebrospinal fluid (CSF) cultures in Pakistan (2011–2015).

Antimicrobials	Antimicrobial group	Prevalence (%) of resistant organisms isolated from patients of different age-groups				Prevalence (%) of resistant organisms isolated from males and female patients			Year-wise prevalence (%) of resistant organisms						Total
		<5 Years	6–18 Years	19–45 Years	P for difference	Female	Male	P for difference	2011	2012	2013	2014	2015	P for trend	
**Nalidixic acid**	Fluoroquinolone	12/16 (75%)*	60/65 (92.3%)	61/64 (95.3%) #	0.03	57/63 (90.5%)	78/85 (91.8%)	NS			25/26 (96.2%)	52/58 (89.7%)	58/64 (90.6%)	NS	135/148 (91.2%)
**Moxifloxacin**	Fluoroquinolone	9/14 (64.3%)	48/77 (62.3%)	57/82 (69.5%)	NS	49/77 (63.6%)	67/100 (67%)	NS	3/9 (33.3%)	1/18 (5.6%)	22/33 (66.7%)	52/58 (89.7%)	38/59 (64.4%)	< .0001	116/177 (65.5%)
**Ciprofloxacin**	Fluoroquinolone	9/16 (56.3%)	49/80 (61.3%)	56/84 (66.7%)	NS	50/81 (61.7%)	66/103 (64.1%)	NS	3/13 (23.1%)	2/20 (10%)	22/33 (66.7%)	52/58 (89.7%)	37/60 (61.7%)	< .0001	116/184 (63%)
**Levofloxacin**	Fluoroquinolone	9/16 (56.3%)	49/82 (59.8%)	57/86 (66.3%)	NS	50/81 (61.7%)	67/107 (62.6%)	NS	3/13 (23.1%)	2/20 (10%)	22/33 (66.7%)	52/58 (89.7%)	38/64 (59.4%)	< .0001	117/188 (62.2%)
**Ofloxacin**	Fluoroquinolone	9/16 (56.3%)	49/82 (59.8%)	57/86 (66.3%)	NS	50/81 (61.7%)	67/107 (62.6%)	NS	3/13 (23.1%)	2/20 (10%)	22/33 (66.7%)	52/58 (89.7%)	38/64 (59.4%)	< .0001	117/188 (62.2%)
**Trimethoprim-Sulphamethoxazole**	Sulphonamides	7/16 (43.8%)	38/77 (49.4%)	39/80 (48.8%)	NS	36/76 (47.4%)	50/101 (49.5%)	NS	10/13 (76.9%)	13/20 (65%)	20/26 (76.9%)	15/57 (26.3%)	28/61 (45.9%)	0.0013 (-)	86/177 (48.6%)
**Ampicillin**	Penicillin	2/16 (12.5%)	9/74 (12.2%)	13/77 (16.9%)	NS	13/75 (17.3%)	11/95 (11.6%)	NS	6/13 (46.2%)	2/6 (33.3%)	10/30 (33.3%)	1/58 (1.7%)	5/63 (7.9%)	< .0001 (-)	24/170 (14.1%)
**Amoxicillin**	Penicillin	2/16 (12.5%)	9/74 (12.2%)	13/77 (16.9%)	NS	13/75 (17.3%)	11/95 (11.6%)	NS	6/13 (46.2%)	2/6 (33.3%)	10/30 (33.3%)	1/58 (1.7%)	5/63 (7.9%)	< .0001 (-)	24/170 (14.1%)
**Amoxicillin-Clavulanic acid**	Penicillin-Inhibitor	1/12 (8.3%)	5/76 (6.6%)	11/79 (13.9%)	NS	10/72 (13.9%)	7/98 (7.1%)	NS	3/13 (23.1%)	5/20 (25%)	6/33 (18.2%)	0/50 (0%)	3/54 (5.6%)	0.0009 (-)	17/170 (10%)
**Ampicillin-Sulbactam**	Penicillin-Inhibitor	1/11 (9.1%)	4/60 (6.7%)	5/54 (9.3%)	NS	6/53 (11.3%)	4/74 (5.4%)	NS	2/3 (66.7%)	1/6 (16.7%)	4/14 (28.6%)	0/50 (0%)	3/54 (5.6%)	0.0003 (-)	10/127 (7.9%)
**Ceftriaxone**	Cephalosporins	0/16 (0%)	1/81 (1.2%)	4/84 (4.8%)	NS	4/81 (4.9%)	1/104 (1%)	NS	1/13 (7.7%)	1/20 (5%)	1/33 (3%)	0/56 (0%)	2/63 (3.2%)	NS	5/185 (2.7%)
**Cefotaxime**	Cephalosporins	0/16 (0%)	1/81 (1.2%)	4/85 (4.7%)	NS	4/80 (5%)	1/106 (0.9%)	NS	1/13 (7.7%)	1/20 (5%)	1/32 (3.1%)	0/57 (0%)	2/64 (3.1%)	NS	5/186 (2.7%)
**Cefixime**	Cephalosporins	0/16 (0%)	0/69 (0%)	3/70 (4.3%)	NS	3/67 (4.5%)	0/91 (0%)	NS		0/6 (0%)	1/32 (3.1%)	0/56 (0%)	2/64 (3.1%)	NS	3/158 (1.9%)
**Cefoperazone**	Cephalosporins	0/16 (0%)	0/74 (0%)	3/74 (4.1%)	NS	3/69 (4.3%)	0/98 (0%)	NS		0/13 (0%)	1/32 (3.1%)	0/58 (0%)	2/64 (3.1%)	NS	3/167 (1.8%)
**Ceftazidime**	Cephalosporins	0/16 (0%)	0/79 (0%)	3/85 (3.5%)	NS	3/81 (3.7%)	0/103 (0%)	NS	0/13 (0%)	0/20 (0%)	1/33 (3%)	0/56 (0%)	2/62 (3.2%)	NS	3/184 (1.6%)
**Cefepime**	Cephalosporins	0/7 (0%)	0/53 (0%)	1/40 (2.5%)	NS	1/44 (2.3%)	0/58 (0%)	NS	0/11 (0%)	0/20 (0%)	0/4 (0%)	0/17 (0%)	1/50 (2%)	NS	1/102 (1%)

Data are number of resistant isolates/total number of isolates (%). Less than 5 isolates were isolated from patients above 45 year of age and these cases were excluded from the age-wise comparison for *S*. Typhi. Denominator varies across cells because all isolates were not tested against the complete set of antimicrobials listed here. Empty cells indicate no cases were tested against the antimicrobial. P-value for difference was calculated by Fisher’s exact test. Bonferroni correction for pairwise comparisons was used to detect differences in percentage of resistant isolates between different age groups.

* indicates age groups less susceptible to resistant infections as compared to the age groups labeled with

#. P-value for trends was calculated by Cochran Armitage test for trends. Two-sided p-value has been reported. P for trend of <0.05 indicate an increasing resistance trend unless P-value is followed by “(-)” which shows a decreasing resistance rates against the antimicrobial in Salmonella enterica serovar Typhi strains. NS: Non significant p-value (i.e. >0.05).

**Table 6:** Temporal, age-wise and gender-wise prevalence of resistant *Staphylococcus aureus* strains isolated from blood and cerebrospinal fluid (CSF) cultures in Pakistan (2011–2015).

Antimicrobials	Antimicrobial group	Prevalence (%) of resistant organisms isolated from patients of different age-groups						Prevalence (%) of resistant organisms isolated from males and female patients			Year-wise prevalence (%) of resistant organisms						Total
		<5	6-18Y	19-45Y	46-65Y	>65Y	P for difference	Female	Male	P for difference	2011	2012	2013	2014	2015	P for Trend	
**Ampicillin**	Penicillin	40/41 (97.6%)	12/13 (92.3%)	48/52 (92.3%)	41/45 (91.1%)	28/29 (96.6%)	NS	64/71 (90.1%)	105/109 (96.3%)	NS	16/19 (84.2%)	17/20 (85%)	33/33 (100%)	46/47 (97.9%)	57/61 (93.4%)	NS	169/180 (93.9%)
**Amoxicillin**	Penicillin	40/41 (97.6%)	12/13 (92.3%)	48/52 (92.3%)	41/45 (91.1%)	28/29 (96.6%)	NS	64/71 (90.1%)	105/109 (96.3%)	NS	16/19 (84.2%)	17/20 (85%)	33/33 (100%)	46/47 (97.9%)	57/61 (93.4%)	NS	169/180 (93.9%)
**Tobramycin**	Aminoglycosides	19/30 (63.3%)	8/12 (66.7%)	24/39 (61.5%)	22/39 (56.4%)	12/24 (50%)	NS	35/59 (59.3%)	50/85 (58.8%)	NS	10/19 (52.6%)	5/7 (71.4%)	11/16 (68.8%)	29/44 (65.9%)	30/58 (51.7%)	NS	85/144 (59%)
**Trimethoprim-Sulphamethoxazole**	Sulphonamides	19/38 (50%)	6/12 (50%)	31/50 (62%)	20/40 (50%)	15/27 (55.6%)	NS	31/65 (47.7%)	60/102 (58.8%)	NS	18/19 (94.7%)	15/20 (75%)	17/27 (63%)	23/47 (48.9%)	18/54 (33.3%)	< .0001 (-)	91/167 (54.5%)
**Erythromycin**	Macrolides	22/38 (57.9%)	6/13 (46.2%)	22/51 (43.1%)	21/44 (47.7%)	17/29 (58.6%)	NS	29/69 (42%)	59/106 (55.7%)	NS	11/18 (61.1%)	12/19 (63.2%)	13/33 (39.4%)	24/44 (54.5%)	28/61 (45.9%)	NS	88/175 (50.3%)
**Azithromycin**	Macrolides	23/39 (59%)	6/13 (46.2%)	22/51 (43.1%)	20/44 (45.5%)	17/29 (58.6%)	NS	28/69 (40.6%)	60/107 (56.1%)	0.046	11/18 (61.1%)	11/19 (57.9%)	13/33 (39.4%)	25/45 (55.6%)	28/61 (45.9%)	NS	88/176 (50%)
**Levofloxacin**	Fluoroquinolone	12/36 (33.3%)	3/12 (25%)	25/48 (52.1%)	23/42 (54.8%)	16/28 (57.1%)	NS	26/68 (38.2%)	53/98 (54.1%)	NS	10/19 (52.6%)	8/20 (40%)	8/19 (42.1%)	23/47 (48.9%)	30/61 (49.2%)	NS	79/166 (47.6%)
**Moxifloxacin**	Fluoroquinolone	12/36 (33.3%)	4/12 (33.3%)	21/40 (52.5%)	21/38 (55.3%)	11/21 (52.4%)	NS	24/61 (39.3%)	45/86 (52.3%)	NS	0/1 (0%)	9/20 (45%)	8/19 (42.1%)	22/46 (47.8%)	30/61 (49.2%)	NS	69/147 (46.9%)
**Amoxicillin-Clavulanic acid**	Penicillin-Inhibitor	20/41 (48.8%)	4/13 (30.8%)	24/52 (46.2%)	19/45 (42.2%)	16/29 (55.2%)	NS	29/71 (40.8%)	54/109 (49.5%)	NS	16/19 (84.2%)	7/20 (35%)	11/33 (33.3%)	23/47 (48.9%)	26/61 (42.6%)	NS	83/180 (46.1%)
**Ofloxacin**	Fluoroquinolone	13/40 (32.5%)	3/13 (23.1%)	25/52 (48.1%)	24/45 (53.3%)	17/29 (58.6%)	NS	27/71 (38%)	55/108 (50.9%)	NS	10/19 (52.6%)	8/20 (40%)	12/33 (36.4%)	23/47 (48.9%)	29/60 (48.3%)	NS	82/179 (45.8%)
**Imipenem**	Carbapenem	20/41 (48.8%)	4/13 (30.8%)	24/52 (46.2%)	19/45 (42.2%)	15/29 (51.7%)	NS	29/71 (40.8%)	53/109 (48.6%)	NS	16/19 (84.2%)	7/20 (35%)	11/33 (33.3%)	23/47 (48.9%)	25/61 (41%)	NS	82/180 (45.6%)
**Meropenem**	Carbapenem	20/41 (48.8%)	4/13 (30.8%)	24/52 (46.2%)	19/45 (42.2%)	15/29 (51.7%)	NS	29/71 (40.8%)	53/109 (48.6%)	NS	16/19 (84.2%)	7/20 (35%)	11/33 (33.3%)	23/47 (48.9%)	25/61 (41%)	NS	82/180 (45.6%)
**Cefaclor**	Cephalosporins	20/41 (48.8%)	4/13 (30.8%)	24/52 (46.2%)	19/45 (42.2%)	15/29 (51.7%)	NS	29/71 (40.8%)	53/109 (48.6%)	NS	16/19 (84.2%)	7/20 (35%)	11/33 (33.3%)	23/47 (48.9%)	25/61 (41%)	NS	82/180 (45.6%)
**Ceftazidime**	Cephalosporins	20/41 (48.8%)	4/13 (30.8%)	24/52 (46.2%)	19/45 (42.2%)	15/29 (51.7%)	NS	29/71 (40.8%)	53/109 (48.6%)	NS	16/19 (84.2%)	7/20 (35%)	11/33 (33.3%)	23/47 (48.9%)	25/61 (41%)	NS	82/180 (45.6%)
**Ceftriaxone**	Cephalosporins	20/41 (48.8%)	4/13 (30.8%)	24/52 (46.2%)	19/45 (42.2%)	15/29 (51.7%)	NS	29/71 (40.8%)	53/109 (48.6%)	NS	16/19 (84.2%)	7/20 (35%)	11/33 (33.3%)	23/47 (48.9%)	25/61 (41%)	NS	82/180 (45.6%)
**Cephalexin**	Cephalosporins	20/41 (48.8%)	4/13 (30.8%)	24/52 (46.2%)	19/45 (42.2%)	15/29 (51.7%)	NS	29/71 (40.8%)	53/109 (48.6%)	NS	16/19 (84.2%)	7/20 (35%)	11/33 (33.3%)	23/47 (48.9%)	25/61 (41%)	NS	82/180 (45.6%)
**Ciprofloxacin**	Fluoroquinolone	14/41 (34.1%)	3/13 (23.1%)	25/52 (48.1%)	23/45 (51.1%)	17/29 (58.6%)	NS	26/71 (36.6%)	56/109 (51.4%)	NS	10/19 (52.6%)	7/20 (35%)	12/33 (36.4%)	23/47 (48.9%)	30/61 (49.2%)	NS	82/180 (45.6%)
**Ampicillin-Sulbactam**	Penicillin-Inhibitor	20/39 (51.3%)	4/13 (30.8%)	16/40 (40%)	17/42 (40.5%)	9/20 (45%)	NS	24/62 (38.7%)	42/92 (45.7%)	NS	0/1 (0%)	7/14 (50%)	10/31 (32.3%)	23/47 (48.9%)	26/61 (42.6%)	NS	66/154 (42.9%)
**Cefepime**	Cephalosporins	18/39 (46.2%)	4/12 (33.3%)	17/43 (39.5%)	15/39 (38.5%)	7/20 (35%)	NS	20/60 (33.3%)	41/93 (44.1%)	NS		2/12 (16.7%)	11/33 (33.3%)	23/47 (48.9%)	25/61 (41%)	NS	61/153 (39.9%)
**Cefoperazone**	Cephalosporins	18/39 (46.2%)	4/12 (33.3%)	17/43 (39.5%)	15/39 (38.5%)	7/20 (35%)	NS	20/60 (33.3%)	41/93 (44.1%)	NS		2/12 (16.7%)	11/33 (33.3%)	23/47 (48.9%)	25/61 (41%)	NS	61/153 (39.9%)
**Cefotaxime**	Cephalosporins	18/39 (46.2%)	4/12 (33.3%)	17/43 (39.5%)	15/39 (38.5%)	7/20 (35%)	NS	20/60 (33.3%)	41/93 (44.1%)	NS		2/12 (16.7%)	11/33 (33.3%)	23/47 (48.9%)	25/61 (41%)	NS	61/153 (39.9%)
**Cephradine**	Cephalosporins	19/40 (47.5%)	4/13 (30.8%)	24/52 (46.2%)	19/45 (42.2%)	15/29 (51.7%)	NS	29/71 (40.8%)	52/108 (48.1%)	NS	16/19 (84.2%)	7/20 (35%)	11/33 (33.3%)	23/47 (48.9%)	24/60 (40%)	NS	81/179 (45.3%)
**Cefixime**	Cephalosporins	18/37 (48.6%)	4/12 (33.3%)	15/37 (40.5%)	14/37 (37.8%)	7/19 (36.8%)	NS	18/52 (34.6%)	40/90 (44.4%)	NS		0/2 (0%)	11/33 (33.3%)	22/46 (47.8%)	25/61 (41%)	NS	58/142 (40.8%)
**Gentamicin**	Aminoglycosides	16/40 (40%)	4/13 (30.8%)	23/51 (45.1%)	17/44 (38.6%)	11/29 (37.9%)	NS	27/71 (38%)	44/106 (41.5%)	NS	11/19 (57.9%)	8/20 (40%)	10/31 (32.3%)	19/47 (40.4%)	23/60 (38.3%)	NS	71/177 (40.1%)
**Cefuroxime**	Cephalosporins	18/39 (46.2%)	4/12 (33.3%)	17/43 (39.5%)	15/39 (38.5%)	7/20 (35%)	NS	20/60 (33.3%)	41/93 (44.1%)	NS		2/12 (16.7%)	11/33 (33.3%)	23/47 (48.9%)	25/61 (41%)	NS	61/153 (39.9%)
**Doxycycline**	Tetracycline	12/34 (35.3%)	4/12 (33.3%)	10/49 (20.4%)	16/43 (37.2%)	11/27 (40.7%)	NS	18/68 (26.5%)	35/97 (36.1%)	NS	8/18 (44.4%)	12/20 (60%)	11/24 (45.8%)	13/47 (27.7%)	9/56 (16.1%)	0.0002 (-)	53/165 (32.1%)
**Clindamycin**	Lincosamide	16/41 (39%)	4/13 (30.8%)	11/52 (21.2%)	13/45 (28.9%)	9/29 (31%)	NS	16/71 (22.5%)	37/109 (33.9%)	NS	2/19 (10.5%)	6/20 (30%)	14/33 (42.4%)	13/47 (27.7%)	18/61 (29.5%)	NS	53/180 (29.4%)
**Amikacin**	Aminoglycosides	8/32 (25%)	1/12 (8.3%)	9/39 (23.1%)	11/41 (26.8%)	2/22 (9.1%)	NS	13/58 (22.4%)	18/88 (20.5%)	NS	8/19 (42.1%)	3/7 (42.9%)	3/16 (18.8%)	7/47 (14.9%)	10/57 (17.5%)	0.0126 (-)	31/146 (21.2%)

Data are number of resistant isolates/total number of isolates (%). Denominator varies across cells because all isolates were not tested against the complete set of antimicrobials listed here Empty cells indicate no cases were tested against the antimicrobial. P-value for difference was calculated by Fisher’s exact test. P-value for trends was calculated by Cochran Armitage test for trends. Two-sided p-value has been reported. P for trend of <0.05 indicate an increasing resistance trend unless P-value is followed by “(-)” which shows a decreasing resistance rates against the antimicrobial. NS: Non significant p-value (i.e. >0.05)

To determine the association between age and sex and trends of antimicrobial resistance, the frequency of isolation of resistance pathogen was calculated by age and sex. The sex-wise comparisons showed that the rate of isolation of resistant gram-negative pathogens were independent of patients’ sex. Evaluation of age-wise resistance trends highlighted that rate of isolation of resistant pathogens is age dependent in that children under 5 years of age had a higher rate of isolation of *E*. *coli* resistant to fluoroquinolones as compared to the elderly (Table 3 and Fig 2). Contrasting trend was observed for amikacin, piperacillin-tazobactam, cefoperazone-sulbactam and carbapenem resistance in *E*. *coli* where we observed that children and young adults had a higher rate of isolation of resistant strains compared to the elderly (Table 3 and Fig 2). Our results have shown a similar trend for cefoperazone-sulbactam resistance in *Acinetobacter* species–children under 5 years of age had higher rate of isolation of cefoperazone-sulbactam resistant *Acinetobacter* species strains as compared to young adults and elderly (Table 4 and Fig 3). We have also reported that *S*. Typhi isolated from adults between 18 to 45 years of age had a higher proportion of Nalidixic acid resistance as compared to those isolated from children under 5 years of age (Table 5 and Fig 4).

We then assessed temporal antimicrobial resistance trends in these pathogens using Cochran Armitage test for trends. Our results show that among gram-negative organisms, *E*. *coli*, demonstrated increasing resistance trend to fluoroquinolones, in that a sharp increase from 50–74% was reported between 2011 and 2015 (Table 3 and Fig 2). Moreover, an increasing resistance trend against cefipime, a fourth-generation cephalosporin, was also observed in *E*. *coli* (Table 3 and Fig 2). While increasing resistance rates were observed for most of the tested antimicrobials, we found decreasing resistance trends against amikacin and gentamicin in *E*. *coli* (Table 3 and Fig 2). The most alarming trends were observed for *Acinetobacter* species (Table 4 and Fig 3), which was found to be resistant to almost all antibiotics. Among these resistance trends, the most notable was the steep increase in carbapenem resistance from 50% in 2011 to 95.5% in 2015 (Table 4 and Fig 3). Between 2011 and 2015 in *S*. Typhi, our results have demonstrated an increasing resistance trend against fluoroquinolones with resistance rates reaching up to 60% in 2015. Additionally, existing trends showed resistance to third and emergence of resistance to fourth generation cephalosporins in *S*. Typhi (Table 5 and Fig 4).

Surprisingly, we did not observe any increasing resistance trend against the tested antimicrobials in *S*. *aureus*. Unlike other pathogens, a decreasing resistance trend to amikacin, doxycycline, and trimethoprim-sulfamethoxazole was observed in *S*. *aureus* (Table 6 and Fig 5). Detailed AMR trends in *S*. *aureus* have been tabulated in Table 6 and shown in Fig 5.

### Co-resistance trends in pathogens isolated from blood and CSF cultures

Multidrug resistance (MDR) has emerged as a major public health problem globally as well as in Pakistan. Co-resistance to multiple drugs emerges with the selection of strains which are resistant to multiple antimicrobials. Clonal expansion of MDR clones is faster as compared to strains resistant to a single antimicrobial [16]. Hence, it is important to identify those antimicrobials, which do not exhibit resistance with any other type of antimicrobials.

To investigate this, antimicrobials belonging to the same class and exhibiting identical resistance profiles in isolates of a given species were merged and entries with missing data were excluded. Chi-square test was used to identify patterns of co-resistance in each pathogen and these patterns are tabulated in Tables 7 and S2–S5. Evaluation of *E*. *coli* showed that resistance against all antimicrobials was significantly associated except β-lactams, aminoglycosides, fluoroquinolones, and tetracycline. Resistance against these four antimicrobials in *E*. *coli* was found to be independent of resistance against other antimicrobials (Table 7 and Fig 2). We then evaluated *Acinetobacter* species and our results showed that cefoperazone-sulbactam resistance is not significantly associated with resistance against aminoglycosides, trimethoprim-sulfamethoxazole and tetracycline (Table 7 and Fig 3). For *S*. Typhi, we only detected a significant co-resistance between nalidixic acid and fluoroquinolones (Table 7 and Fig 4). Analysis of *S*. *aureus* showed that penicillin resistance was not associated with resistance against any other antimicrobials. Furthermore, doxycycline resistance in *S*. *aureus* was found to be independent of resistance to all antimicrobials except macrolides and tobramycin (Table 7 and Fig 5). The co-resistance proportions, p-values, and associated odds ratios for all tested antimicrobials are provided in Tables 7 and S2–S5. The co-resistance trends for *E*. *coli*, *Acinetobacter* species, *S*. Typhi, and *S*. *aureus* are presented in Figs 2–5.

**Table 7:** Selected co-resistance trends in pathogens isolated from blood and cerebrospinal fluid (CSF) cultures in Pakistan (2011–2015).

Pathogen	Antimicrobial 1	Number of isolates resistant to antimicrobial 1 & 2/ Number of isolates resistant to antimicrobial 1 (%)	Number of isolates resistant to antimicrobial 1 & 2/ Number of isolates resistant to antimicrobial 2 (%)	P for difference	Odds ratio 95% CI)	Antimicrobial 2
***Acinetobacter***	**Cefoperazone-sulbactam**	54/ 54 (100)	54/ 104 (51.9)	0.118	NA	**Piperacillin-tazobactam**
		52/ 54 (96.3)	52/ 100 (52)	0.2702	3.25 (0.63–16.88)	**Amikacin**
		42/ 54 (77.8)	42/ 79 (53.2)	0.2777	1.61 (0.68–3.8)	**Tobramycin**
		53/ 54 (98.1)	53/ 103 (51.5)	0.3632	4.24 (0.46–39.24)	**Gentamicin**
		29/ 54 (53.7)	29/ 55 (52.7)	0.5636	1.25 (0.59–2.66)	**Doxycycline**
		48/ 54 (88.9)	48/ 96 (50)	1	1 (0.3–3.32)	**Trimethoprim-sulphamethoxazole**
	**Cephalosporin**	79/ 106 (74.5)	79/ 79 (100)	0.0703	NA	**Tobramycin**
		103/ 106 (97.2)	103/ 104 (99)	0.073	34.33 (1.71–689.68)	**Piperacillin-tazobactam**
		99/ 106 (93.4)	99/ 100 (99)	0.1433	14.14 (0.8–250.9)	**Amikacin**
		55/ 106 (51.9)	55/ 55 (100)	0.2385	NA	**Doxycycline**
		54/ 106 (50.9)	54/ 54 (100)	0.4953	NA	**Cefoperazone-sulbactam**
	**Carbapenem**	78/ 104 (75)	78/ 79 (98.7)	0.0583	9 (0.9–90.33)	**Tobramycin**
		103/ 104 (99)	103/ 106 (97.2)	0.073	34.33 (1.71–689.68)	**Cephalosporin**
		54/ 104 (51.9)	54/ 54 (100)	0.118	NA	**Cefoperazone-sulbactam**
		54/ 104 (51.9)	54/ 55 (98.2)	0.3587	3.24 (0.33–32.18)	**Doxycycline**
	**Piperacillin-tazobactam**	78/ 104 (75)	78/ 79 (98.7)	0.0583	9 (0.9–90.33)	**Tobramycin**
		54/ 104 (51.9)	54/ 55 (98.2)	0.3587	3.24 (0.33–32.18)	**Doxycycline**
	**Doxycycline**	51/ 55(92.7)	51/ 96(53.1)	0.196	2.27(0.64–8.03)	**Trimethoprim-sulphamethoxazole**
	**Tobramycin**	72/ 79(91.1)	72/ 96(75)	0.2984	2.14(0.62–7.38)	**Trimethoprim-sulphamethoxazole**
***Escherichia coli***	**Carbapenem**	3/ 6 (50)	3/ 133 (2.3)	0.1118	0.25 (0.05–1.32)	**Doxycycline**
		6/ 6 (100)	6/ 119 (5)	0.181	NA	**Tobramycin**
		5/ 6 (83.3)	5/ 96 (5.2)	0.2367	3.96 (0.45–34.62)	**Gentamicin**
		6/ 6 (100)	6/ 137 (4.4)	0.3604	NA	**4**^**th**^ **generation cephalosporin**
		6/ 6 (100)	6/ 139 (4.3)	0.3732	NA	**Trimethoprim-sulphamethoxazole**
		6/ 6 (100)	6/ 144 (4.2)	0.5935	NA	**3**^**rd**^ **generation cephalosporin**
		6/ 6 (100)	6/ 145 (4.1)	0.5958	NA	**Aztreonam**
		6/ 6 (100)	6/ 145 (4.1)	0.5958	NA	**Aztreonam**
		6/ 6 (100)	6/ 153 (3.9)	0.6462	NA	**Penicillin**
		6/ 6 (100)	6/ 153 (3.9)	0.6462	NA	**Penicillin & β-lactamase inhibitor**
		6/ 6 (100)	6/ 154 (3.9)	0.6572	NA	**2**^**nd**^ **generation cephalosporin**
		5/ 6 (83.3)	5/ 128 (3.9)	1	1.63 (0.18–14.33)	**Fluoroquinolone**
	**Cefoperazone-sulbactam**	12/ 15 (80)	12/ 96 (12.5)	0.0575	3.33 (0.9–12.28)	**Gentamicin**
		15/ 15 (100)	15/ 137 (10.9)	0.0772	1 (17–120)	**4**^**th**^ **generation cephalosporin**
		15/ 15 (100)	15/ 144 (10.4)	0.1308	NA	**3**^**rd**^ **generation cephalosporin**
		14/ 15 (93.3)	14/ 139 (10.1)	0.3139	3.25 (0.41–25.7)	**Trimethoprim-sulphamethoxazole**
		15/ 15 (100)	15/ 153 (9.8)	0.3657	1 (17–136)	**Penicillin & β-lactamase inhibitor**
		15/ 15 (100)	15/ 154 (9.7)	0.3665	1 (17–137)	**2**^**nd**^ **generation cephalosporin**
		13/ 15 (86.7)	13/ 128 (10.2)	0.3665	2.2 (0.48–10.2)	**Fluoroquinolone**
		14/ 15 (93.3)	14/ 145 (9.7)	0.4817	2.46 (0.31–19.61)	**Aztreonam**
		11/ 15 (73.3)	11/ 133 (8.3)	0.7408	0.72 (0.22–2.42)	**Doxycycline**
	**Piperacillin-tazobactam**	17/ 17 (100)	17/ 144 (11.8)	0.0795	NA	**3**^**rd**^ **generation cephalosporin**
		13/ 17 (76.5)	13/ 96 (13.5)	0.0843	2.7 (0.84–8.66)	**Gentamicin**
		17/ 17 (100)	17/ 145 (11.7)	0.1351	1 (152–1)	**Aztreonam**
		16/ 17 (94.1)	16/ 139 (11.5)	0.2094	3.77 (0.48–29.61)	**Trimethoprim-sulphamethoxazole**
		17/ 17 (100)	17/ 153 (11.1)	0.2284	1 (137–17)	**Penicillin & β-lactamase inhibitor**
		17/ 17 (100)	17/ 154 (11)	0.2374	1 (137–0)	**2**^**nd**^ **generation cephalosporin**
		15/ 17 (88.2)	15/ 128 (11.7)	0.2494	2.59 (0.57–11.83)	**Fluoroquinolone**
		14/ 17 (82.4)	14/ 133 (10.5)	0.7702	1.29 (0.35–4.77)	**Doxycycline**
	**Amikacin**	15/ 22(68.2)	15/ 133(11.3)	0.2613	0.53 (0.2–1.41)	**Doxycycline**
		21/ 22(95.5)	21/ 153(13.7)	0.4866	2.39 (0.3–19.02)	**Penicillin & β-lactamase inhibitor**
		21/ 22(95.5)	21/ 154(13.6)	0.6957	2.21 (0.28–17.7)	**2**^**nd**^ **generation cephalosporin**
		16/ 22(72.7)	16/ 128(12.5)	0.7238	0.83 (0.3–2.29)	**Fluoroquinolone**
		17/ 22(77.3)	17/ 137(12.4)	0.7703	0.77 (0.26–2.25)	**4**^**th**^ **generation cephalosporin**
		18/ 22(81.8)	18/ 139(12.9)	1	0.97 (0.3–3.09)	**Trimethoprim-sulphamethoxazole**
		19/ 22(86.4)	19/ 145(13.1)	1	1.06 (0.29–3.88)	**Aztreonam**
		19/ 22(86.4)	19/ 144(13.2)	1	1.11 (0.3–4.09)	**3**^**rd**^ **generation cephalosporin**
	**Gentamicin**	78/ 96 (81.3)	78/ 133 (58.6)	0.3528	1.42 (0.68–2.97)	**Doxycycline**
***Salmonella enterica* serovar Typhi**	**Penicillin**	4/5 (80)	4/ 49 (8.1)	0.172	5.24 (0.567–48.55)	**Trimethoprim-sulphamethoxazole**
		4/5 (80)	4/ 100 (4)	0.356	0.333 (.033–3.348)	**Nalidixic acid**
		4/5 (80)	4/ 86 (4.65)	1	1.073 (0.114–10.93)	**Fluoroquinolone**
	**Trimethoprim-sulphamethoxazole**	46/49 (93.8)	46/100 (46)	0.51	1.704 (0.403–7.195)	**Nalidixic acid**
		39/49 (79.6)	39/86 (45.3)	1	1.079 (.427–2.727)	**Fluoroquinolone**
***Staphylococcus aureus***	**Doxycycline**	7/ 24 (29.2)	7/ 16 (43.8)	0.0502	2.97 (0.97–9.14)	**Amikacin**
		11/ 24 (45.8)	11/ 31 (35.5)	0.0852	2.28 (0.88–5.92)	**Clindamycin**
		15/ 24 (62.5)	15/ 48 (31.3)	0.1273	2.07 (0.8–5.33)	**Macrolides**
		13/ 24 (54.2)	13/ 44 (29.5)	0.2935	1.64 (0.65–4.14)	**β-lactam (except penicillin)**
		11/ 24 (45.8)	11/ 37 (29.7)	0.3475	1.56 (0.61–3.98)	**Gentamicin**
		12/ 24 (50)	12/ 47 (25.5)	0.8179	1.11 (0.44–2.8)	**Fluoroquinolone**
	**Trimethoprim-sulphamethoxazole**	25/ 44 (56.8)	25/ 48 (52.1)	0.1612	1.77 (0.79–3.96)	**Macrolides**
		8/ 44 (18.2)	8/ 16 (50)	0.6538	1.28 (0.44–3.74)	**Amikacin**
	**Penicillin**	58/ 95 (61.1)	58/ 59 (98.3)	0.5613	3.14 (0.27–35.81)	**Tobramycin**

Data are number of isolates resistant to both antimicrobials / number of isolates resistant to either antimicrobial 1 (in the case of R1) or antimicrobial 2 (in the case of R1) (%). P-value for difference was calculated using Chi-square test. Odds-ratio was calculated using binary logistic regression and is listed with 95% confidence interval (95% CI). Two-sided p-value has been reported.

## Discussion

Antimicrobial resistance (AMR) has emerged as a major public health concern in both developing and developed countries. Continuous surveillance of AMR has been recommended by World Health Organization (WHO) as a necessary step for controlling emergence of resistance as well as infections caused by resistant pathogens [17]. Despite this urgent need to investigate AMR trends, only a handful of studies till date have reported resistance trends in pathogens isolated from blood and CSF cultures in Pakistan [18, 19]. The current study stands to fill this gap in knowledge of AMR patterns and trends in these bacterial pathogens at a national scale. For that, we undertook a retrospective analysis of AMR of pathogens isolated from clinical specimens of blood and CSF. To the best of our knowledge, this study is the first of its kind in Pakistan providing both demographic and temporal AMR trends in major pathogens from blood and CSF cultures. Previously published results have been limited by the number of participants the sample size and analysis. Data presented in this report provides a more in-depth analysis of antimicrobial resistance. While most of the previous studies were cross-sectional presenting resistance at single time point, our long-term analysis presents alterations in resistance patterns over five years.

Our results show that CoNS was the most frequently isolated pathogen from invasive isolates which is consistent with previous studies in southeast Asia [20]. However, as we did not have information on the clinical manifestation of these cases and they may have been skin contaminants, we removed CoNS from the downstream analysis. Two of the most probable reasons for high rate of isolation of CoNS in Pakistan could be poor clinical sample management and presence of contaminants on surfaces [21].

In accordance with international reports, our data also reflects emergence of antimicrobial resistance in Pakistan, where pathogens are resistant to most commonly used antimicrobials [1, 3, 22]. Our analysis showed a wide distribution of resistant pathogen over 44 cities of Pakistan, however most of our observations were from the province of Punjab, which could be because the facility is primarily located in Punjab. No significant association was observed when comparisons were made by sex, indicating that both male and female are equally susceptible to infections caused by resistant pathogens. Similarly, no significant association between a sex and a specific pathogen was observed. However, we did observe a significant association between rates of isolation of resistant pathogens with age, younger children and elderly were at a higher risk of infections by drug resistant pathogens. This observation is in line with previous findings where infections with resistant pathogens were found to be associated with a children and elderly [23–25]. The most likely explanation for these observations is the increase rates of infections in both elderly and children. The observed increase susceptibility to infections in children and elderly is due to poor immune responses. As a result antimicrobials are frequently and empirically used in both children and elderly resulting in increased rates of infections by resistant pathogens.

Upper respiratory tract infections and gastrointestinal tract infections are the most frequent cause of doctors visit for children and elderly in Pakistan. Such infections are most often treated empirically by broad spectrum antibiotics without performing culture sensitivity, leading to development of resistance in both, the resident bacteria and infecting pathogen. Comparison of rates of isolation of different invasive pathogens indicated an increase in rate of isolation of *S*. *maltophilia* between 2013 and 2014. *Stenotrophomonas maltophilia*, previously known as *Pseudomonas maltophilia* primarily cause infections in immune compromised individuals. Together with *Pseudomonas aeruginosa* and *Acinetobacter baumannii*, *S*.*maltophilia* is an important cause of healthcare-associated infections. Moreover, *A*. *baumannii* and *S*. *maltophilia* have also been shown to co-exist, causing multi drug resistant polymicrobial infections in hospitalized patients. Most common source of these infections are contaminated endoscopes and dialysis machines where *S*. *maltophilia* and *A*. *baumannii* has been shown to form biofilms [21, 26–29].

Another salient observation of our study is the rapid rise of carbapenems resistance among *Acinetobacter* species (CRA) (from 50% to 95% between 2011 and 2015) in Pakistan. This is in line with earlier CRA trends in Southeast Asian countries [30, 31]. This increase is concomitant with the two-fold increase in carbapenem usage in Pakistan over the last decade [32, 33]. It is critical to note that carbapenems are one of the few last resort broad-spectrum antibiotics recommended for treatment of sepsis in Pakistan [34] and emergence of CRA has further limited the therapeutic options. Only doxycycline and cefoperazone-sulbactam remain effective against many of the *Acinetobacter* strains (Table 4). The persistent susceptibility to doxycycline may be attributed to its limited usage in *Acinetobacter* infections treatment due to its bacteriostatic nature [35]. Further, while resistance against cefoperazone-sulbactam was found to be on the rise (Table 4), a wider acquisition of resistance against it might require more time. Additionally, *Acinetobacter* is an environmental pathogen found in the soil, the acquisition of resistance therefore suggests inappropriate disposal of antimicrobials. To further explain this, it is likely that unused or expired antimicrobials are being disposed of in a way that they are available to environmental organisms resulting in emergence of resistance in such organism [36, 37]. This situation provides policymakers with an opportunity to legislate regulated usage of carbapenem, cefoperazone-sulbactam, and doxycycline in the country, and ensure proper disposal of such antimicrobials from hospital systems. Developed countries like the US and the UK have resorted to using colistin, polymixin B, and tigecycline for the treatment of CRA infections [38, 39]. Susceptibility data against these antibiotics were not accounted for in this study. Future studies should highlight the susceptibility patterns of *Acinetobacter* against colistin, polymixin B, and tigecycline.

Another important finding of this study is the presence of third and fourth generation cephalosporins resistance in *S*. Typhi. Recent studies have reported an incidence of third generation resistant *S*. Typhi infections in Pakistan and neighboring countries [40, 41]. These antimicrobials are the drugs of choice for the empirical treatment of these infections [42]. This suggests that treatment of these infections with cephalosporins will become ineffective. Case studies from Canada and the US have shown that these infections can successfully be treated with carbapenems and ceftriaxone-azithromycin combination [43]. However, it is highly likely that *S*. Typhi will acquire resistance against these antimicrobials in the short term. In the light of this, WHO has recommended large scale implementation of Typbar-TCV, a typhoid vaccine, for the containment of *S*. Typhi infections in high risk regions [42, 44]. Improved personal hygiene, handwashing, availability of clean drinking water, and well cooked food lowers the risk of *S*. Typhi infections by 20% [42, 45]. The government should actively work towards implementing these suggestions to limit the spread of drug resistant *S*. Typhi infections.

While AMR was rising in other major pathogens, we unexpectedly found decreasing resistance rates against few major antimicrobials in *S*. *aureus*. This is in line with the reports on incidence of antimicrobial resistant *S*. *aureus* infections worldwide [46, 47]. While the exact reason for this decrease is yet to be identified, studies have suggested that it may be due to improved infection control practices and improved antibiotic prescription guidelines [46]. The ability of *S*. *aureus* to excise resistance markers, out of its genome, in the absence of antimicrobials may have played a role in the decreasing resistance as well. Excision of resistance markers is associated with reduced metabolic cost and overall increase in the bacterial fitness [46, 48, 49]. While the current resistance levels in *S*. *aureus* are low, continuous surveillance is required to keep track of emerging resistance trends in this pathogen.

### Study limitation

This study is limited in that two provinces, namely Baluchistan and Gilgit-Baltistan, were not represented. The combined population of these two provinces constitutes less than 10% of the population of and our sample was representative of two large provinces namely Punjab and Sindh where 90% of Pakistani population resides. Therefore we can comfortably state that this is the representative sample of the country. However, this does not exclude the need for performing similar studies in areas such as Gilgit-Baltistan and Baluchistan [50]. However, excessing data from these regions is difficult and adds to the limitations of our study. Next, while our results have shown CoNS to be the most prevalent bacterial pathogen isolated from the blood and CSF cultures, this can be explained by CoNS being a common skin contaminant. For the purpose of this study, we have treated CoNS as a contaminant and not reported resistance trends in CoNS. However, further studies need to be carried to determine the role of CoNS as a potential bacteremia pathogen. Due to the unavailability of the patient characteristics we were also limited in our analysis of the impact of comorbid conditions on the susceptibility to invasive infections by resistant bacteria. Data regarding antimicrobial prescription practices was also lacking, preventing us to determine the impact of prescription practices in a geographic region on emergence of resistance. Since a significant proportion of our patient population was from Punjab and KPK, our analysis by geographic region was limited by lack of power. Lastly, susceptibility data on last resort antibiotics, including colistin, polymixin B, and linezolid, was not available. Susceptibility patterns of invasive pathogens to these antibiotics still need to be carried out.

## Conclusions

In this study we set out to determine resistance patterns in pathogens isolated from blood and CSF cultures. We found that resistance has been at rise for several of these pathogens. Highest resistance rates were observed in *Acinetobacter* species against all tested antimicrobials including carbapenems. Resistance against 3^rd^ and 4^th^ generation cephalosporins has been reported in *S*. Typhi during the study period. Policy makers should prioritize and expedite implementation of infection control practices and antimicrobial stewardship in the country to control the emerging threat of AMR to public health.

## Supporting information

S1 Table Distribution of bacterial isolates from blood and cerebrospinal fluid (CSF) cultures from different cities. (DOCX)

S2 Table Co-resistance patterns in *Escherichia coli*. (DOCX)

S3 Table Co-resistance patterns in *Acinetobacter*. (DOCX)

S4 Table Co-resistance patterns in *Salmonella enterica* serovar Typhi. (DOCX)

S5 Table Co-resistance patterns in *Staphylococcus aureus*. (DOCX)

S6 Table Complete dataset. (XLSX)
