# Supplementary material for: Trends in antimicrobial resistance amongst pathogens isolated from blood and cerebrospinal fluid cultures in Pakistan (2011-2015): A retrospective cross-sectional study
Source: PLoS One. 2021 Apr 26;16(4):e0250226. doi: 10.1371/journal.pone.0250226 (PMC8075205; doi:10.1371/journal.pone.0250226)
Supplement: S1 Table — (DOCX) [file pone.0250226.s001.docx]

**S1 Table. Distribution of bacterial isolates from Blood and Cerebrospinal Fluid (CSF) cultures from different cities**

| **City of sample collection** | **Bacterial Isolates obtained from Blood and Cerebrospinal Fluid cultures** |
| --- | --- |
| Abbottabad | 254 (8.3) |
| Alipur Chatta | 2 (0.1) |
| Bahawalnagar | 1 (0) |
| Bahawalpur | 22 (0.7) |
| Bhakkar | 4 (0.1) |
| Bure Wala | 2 (0.1) |
| Chichawatni | 1 (0) |
| DG Khan | 4 (0.1) |
| Faisalabad | 559 (18.2) |
| Gujranwala | 26 (0.8) |
| Gujrat | 2 (0.1) |
| Haripur Hazara | 6 (0.2) |
| Islamabad | 4 (0.1) |
| Jhang | 1 (0) |
| Karachi | 4 (0.1) |
| Kasur | 4 (0.1) |
| Khanewal | 1 (0) |
| Khushab | 15 (0.5) |
| Lahore | 1855 (60.5) |
| Layyah | 2 (0.1) |
| Mansehra | 1 (0) |
| Multan | 40 (1.3) |
| Nankana | 4 (0.1) |
| Narowal | 3 (0.1) |
| Okara | 3 (0.1) |
| Pakpattan | 2 (0.1) |
| Peshawar | 78 (2.5) |
| Quetta | 1 (0) |
| Rahim Yar Khan | 22 (0.7) |
| Rawalpindi | 45 (1.5) |
| Renala Khurd | 8 (0.3) |
| Sahiwal | 12 (0.4) |
| Sargodha | 16 (0.5) |
| Sheikhupura | 4 (0.1) |
| Sialkot | 13 (0.4) |
| Ghotki | 4 (0.1) |
| Khanpur Mahar | 1 (0) |
| Pano Aqil | 2 (0.1) |
| Shandadkot | 2 (0.1) |
| Larkana | 5 (0.2) |
| Jacobabad | 6 (0.2) |
| Toba | 8 (0.3) |
| Vehari | 15 (0.5) |
| No information | 4 (0.1) |
| **Total** | **3068** |
