# Supplementary material for: Trends in antimicrobial resistance amongst pathogens isolated from blood and cerebrospinal fluid cultures in Pakistan (2011-2015): A retrospective cross-sectional study
Source: PLoS One. 2021 Apr 26;16(4):e0250226. doi: 10.1371/journal.pone.0250226 (PMC8075205; doi:10.1371/journal.pone.0250226)
Supplement: S2 Table — (DOCX) [file pone.0250226.s002.docx]

**S2 Table. Co-resistance patterns in *Escherichia coli***

| **Antimicrobial** | **Variables** | **Piperacillin-tazobactam** | **Cefoperazone-sulbactam** | **Trimethoprim-sulphamethoxazole** | **Fluoroquinolone** | **Doxycycline** | **Tobramycin** | **Gentamicin** | **Amikacin** | **1^st^ Generation cephalosporin** | **3^rd^ Generation cephalosporin** | **4^th^ Generation cephalosporin** | **2^nd^ Generation cephalosporin** | **Penicillin & β-lactamase Inhibitors** | **Aztreonam** |
| --- | --- | --- | --- | --- | --- | --- | --- | --- | --- | --- | --- | --- | --- | --- | --- |
| **Carbapenem** | **R1**  **n/N (%)** | 6/6  (100) | 6/6  (100) | 6/ 6  (100) | 5/ 6  (83.3) | 3/ 6  (50) | 6/ 6  (100) | 5/ 6  (83.3) | 4/6  (66.7) | 6/6  (100) | 6/ 6  (100) | 6/ 6(100) | 6/ 6  (100) | 6/ 6  (100) | 6/ 6  (100) |
|  | **R2**  **n/N (%)** | 6/11 (54.5) | 6/15  (40) | 6/ 139  (4.3) | 5/ 128  (3.9) | 3/ 133  (2.3) | 6/ 119  (5) | 5/ 96  (5.2) | 4/22 (18.2) | 6/ 169  (3.6) | 6/ 144  (4.2) | 6/ 137(4.4) | 6/ 154  (3.9) | 6/ 153  (3.9) | 6/ 145  (4.1) |
|  | **P-value** | <0.005 | <0.005 | 0.3732 | 1 | 0.1118 | 0.181 | 0.2367 | <0.005 | NA | 0.5935 | 0.3604 | 0.6572 | 0.6462 | 0.5958 |
|  | **Odds ratio**  **(95% CI)** | NA | NA | NA | 1.63  (0.2-14.3) | 0.25  (0.05-1.3) | NA | 3.96  (0.5-34.6) | 16.1  (2.7-94.3) | NA | NA | NA | NA | NA | NA |
| **Aztreonam** | **R1**  **n/N (%)** | 17/ 145  (11.7) | 14/ 145  (9.7) | 132/145  (91) | 120/145 (82.8) | 121/145 (83.4) | 111/145 (76.6) | 90/145 (62.1) | 19/ 145  (13.1) | 145/145  (100) | 141/145 (97.2) | 136/145 (93.8) | 145/145 (100) | 145/145 (100) |  |
|  | **R2**  **n/N (%)** | 17/17 (100) | 14/15  (93.3) | 132/139  (95) | 120/128 (93.8) | 121/133 (91) | 111/119 (93.3) | 90/96 (93.8) | 19/ 22  (86.4) | 145/ 169  (85.8) | 141/144 (97.9) | 136/137 (99.3) | 145/154 (94.2) | 145/153 (94.8) |  |
|  | **P-value** | 0.1351 | 0.4817 | <0.005 | <0.005 | <0.005 | 0 | <0.005 | 1 | NA | <0.005 | <0.005 | <0.005 | <0.005 |  |
|  | **Odds ratio**  **(95% CI)** | NA | 2.46  (0.31-19.61) | 24.66  (8.64-70.36) | 9.6  (3.7-24.87) | 5.04  (2-12.55) | 6.53  (2.6-16.58) | 4.91  (1.8-13.12) | 1.06  (0.29-3.88) | NA | 246.8  (51.6-1180.8) | 347.56  (42-2874.51) | NA | NA |  |
| **Penicillin & β-lactamase Inhibitors** | **R1**  **n/N (%)** | 17/ 153  (11.1) | 15/ 153  (9.8) | 134/153 (87.6) | 124/153 (81) | 126/153 (82.4) | 117/153 (76.5) | 94/153 (61.4) | 21/ 153  (13.7) | 153/153  (100) | 144/153 (94.1) | 137/153 (89.5) | 152/153 (99.3) |  |  |
|  | **R2**  **n/N (%)** | 17/17 (100) | 15/ 15  (100) | 134/139 (96.4) | 124/128 (96.9) | 126/133 (94.7) | 117/119 (98.3) | 94/96 (97.9) | 21/ 22  (95.5) | 153/ 169  (90.5) | 144/144 (100) | 137/137 (100) | 152/154 (98.7) |  |  |
|  | **P-value** | 0.2284 | 0.3657 | <0.005 | <0.005 | <0.005 | <0.005 | <0.005 | 0.4866 | NA | <0.005 | <0.005 | <0.005 |  |  |
|  | **Odds ratio**  **(95% CI)** | NA | NA | 15.5  (94.9-49.5) | 12.83  (3.9-42.7) | 6  (2.-17.5) | 22.75  (4.9-104.8) | 11.15  (2.4-50.8) | 2.39  (0.3-19) | NA | NA | NA | 1064  (90.-12479) |  |  |
| **2^nd^ Generation cephalosporin** | **R1**  **n/N (%)** | 17/ 154  (11) | 15/ 154  (9.7) | 136/154 (88.3) | 124/154 (80.5) | 127/154 (82.5) | 117/154 (76) | 95/154 (61.7) | 21/ 154  (13.6) | 154/154 (100) | 144/154 (93.5) | 137/154  (89) |  |  |  |
|  | **R2**  **n/N (%)** | 17/ 17  (100) | 15/ 15  (100) | 136/139 (97.8) | 124/128 (96.9) | 127/133 (95.5) | 117/119 (98.3) | 95/96  (99) | 21/ 22  (95.5) | 154/169 (91.1) | 144/144  (100) | 137/137 (100) |  |  |  |
|  | **P-value** | 0.2374 | 0.3665 | <0.005 | <0.005 | <0.005 | <0.005 | <0.005 | 0.6957 | NA | <0.005 | <0.005 |  |  |  |
|  | **Odds ratio**  **(95% CI)** | NA | NA | 30.22  (7.8-117.4) | 11.37  (3.4-38.2) | 7.06  (2.3-21.5) | 20.55  (4.3-95.3) | 22.54  (2.9-175.9) | 2.21  (0.28-17.7) | NA | NA | NA |  |  |  |
| **4^th^ Generation cephalosporin** | **R1**  **n/N (%)** | 17/120 (14.2) | 15/137 (10.9) | 125/137 (91.2) | 116/137 (84.7) | 113/137 (82.5) | 105/137 (76.6) | 85/137 (62) | 17/ 13 (12.4) | 137/137 (100) | 137/137  (100) |  |  |  |  |
|  | **R2**  **n/N (%)** | 17/17 (100) | 15/15  (100) | 125/139 (89.9) | 116/128 (90.6) | 113/133 (85) | 105/119 (88.2) | 85/96 (88.5) | 17/ 22 (77.3) | 137/169 (81.1) | 137/144 (95.1) |  |  |  |  |
|  | **P-value** | 0.045 | 0.0772 | <0.005 | <0.005 | 0.017 | <0.005 | 0.006 | 0.7703 | NA | <0.005 |  |  |  |  |
|  | **Odds ratio**  **(95% CI)** | NA | NA | 13.39  (5.36-33.47) | 9.2  (3.92-21.61) | 2.83  (1.21-6.55) | 4.22  (1.89-9.41) | 3.12  (1.39-6.99) | 0.77 (0.26-2.25) | NA | 4.57  (2.38-8.8) |  |  |  |  |
| **3^rd^ Generation cephalosporin** | **R1**  **n/N (%)** | 17/ 144  (11.8) | 15/ 144  (10.4) | 130/144 (90.3) | 120/144 (83.3) | 119/144 (82.6) | 110/144 (76.4) | 89/144 (61.8) | 19/144 (13.2) | 144/144 (100) |  |  |  |  |  |
|  | **R2**  **n/N (%)** | 17/17 (100) | 15/15  (100) | 130/139 (93.5) | 120/128 (93.8) | 119/133 (89.5) | 110/119 (92.4) | 89/96 (92.7) | 19/22 (86.4) | 144/169 (85.2) |  |  |  |  |  |
|  | **P-value** | 0.0795 | 0.1308 | <0.005 | <0.005 | 0.005 | <0.005 | <0.005 | 1 | NA |  |  |  |  |  |
|  | **Odds ratio**  **(95% CI)** | NA | NA | 16.51  (6.16-44.22) | 10.63  (4.12-27.41) | 3.74  (1.52-9.2) | 5.75  (2.33-14.19) | 4.16  (1.63-10.6) | 1.11 (0.3-4.09) | NA |  |  |  |  |  |
| **1^st^ Generation cephalosporin** | **R1**  **n/N (%)** | 17/169 (10.1) | 15/169  (8.9) | 139/169 (82.2) | 128/169 (75.7) | 133/169 (78.7) | 119/169 (70.4) | 96/169 (56.8) | 22/ 169 (13) |  |  |  |  |  |  |
|  | **R2**  **n/N (%)** | 17/17 (100) | 15/15 (100) | 139/139 (100) | 128/128 (100) | 133/133 (100) | 119/119 (100) | 96/96 (100) | 22/ 22 (100) |  |  |  |  |  |  |
|  | **P-value** | NA | NA | NA | NA | NA | NA | NA | NA |  |  |  |  |  |  |
|  | **Odds ratio**  **(95% CI)** | NA | NA | NA | NA | NA | NA | NA | NA |  |  |  |  |  |  |
| **Amikacin** | **R1**  **n/N (%)** | 8/22 (36.4) | 8/22  (36.4) | 18/22  (81.8) | 16/22 (72.7) | 15/22 (68.2) | 22/22  (100) | 22/22 (100) |  |  |  |  |  |  |  |
|  | **R2**  **n/N (%)** | 8/17  (47) | 8/15  (53.3) | 18/139  (12.9) | 16/128 (12.5) | 15/133 (11.3) | 22/119 (18.5) | 22/96 (22.9) |  |  |  |  |  |  |  |
|  | **P-value** | <0.005 | <0.005 | 1 | 0.7238 | 0.2613 | <0.005 | <0.005 |  |  |  |  |  |  |  |
|  | **Odds ratio**  **(95% CI)** | 8.76  (2.9-26.31) | 11.43  (3.6-36.22) | 0.97  (0.3-3.09) | 0.83  (0.3-2.29) | 0.5  3(0.2-1.41) | NA | 1.97  (1.7-2.33) |  |  |  |  |  |  |  |
| **Gentamicin** | **R1**  **n/N (%)** | 13/96 (13.5) | 12/ 96  (12.5) | 85/96  (88.5) | 84/96 (87.5) | 78/96 (81.3) | 96/96  (100) |  |  |  |  |  |  |  |  |
|  | **R2**  **n/N (%)** | 13/17 (76.5) | 12/ 15  (80) | 85/139  (61.2) | 84/128 (65.6) | 78/133 (58.6) | 96/119 (80.7) |  |  |  |  |  |  |  |  |
|  | **P-value** | 0.121 | 0.0575 | 0.016 | <0.005 | 0.3528 | <0.005 |  |  |  |  |  |  |  |  |
|  | **Odds ratio**  **(95% CI)** | 2.7  (0.84-8.66) | 3.33  (0.9-12.28) | 2.72  (1.2-6.16) | 4.61  (2.15-9.92) | 1.42  (0.68-2.97) | NA |  |  |  |  |  |  |  |  |
| **Tobramycin** | **R1**  **n/N (%)** | 16/119 (13.4) | 15/119 (12.6) | 106/119 (89.1) | 106/119 (89.1) | 99/119 (83.2) |  |  |  |  |  |  |  |  |  |
|  | **R2**  **n/N (%)** | 16/17 (94.1) | 15/15  (100) | 106/139 (76.3) | 106/128 (82.8) | 99/133 (74.4) |  |  |  |  |  |  |  |  |  |
|  | **P-value** | 0.024 | 0.014 | <0.005 | <0.005 | 0.039 |  |  |  |  |  |  |  |  |  |
|  | **Odds ratio**  **(95% CI)** | 7.61  (0.98-59.05) | NA | 4.2  (1.85-9.55) | 10.38  (4.65-23.15) | 2.33  (1.09-5) |  |  |  |  |  |  |  |  |  |
| **Doxycycline** | **R1**  **n/N (%)** | 14/133 (10.5) | 11/133  (8.3) | 118/133 (88.7) | 108/133 (81.2) |  |  |  |  |  |  |  |  |  |  |
|  | **R2**  **n/N (%)** | 14/17 (82.4) | 11/15  (73.3) | 118/139 (84.9) | 108/128 (84.4) |  |  |  |  |  |  |  |  |  |  |
|  | **P-value** | 0.7702 | 0.7408 | <0.005 | <0.005 |  |  |  |  |  |  |  |  |  |  |
|  | **Odds ratio**  **(95% CI)** | 1.29  (0.35-4.77) | 0.72  (0.22-2.42) | 5.61  (2.39-13.19) | 3.46  (1.57-7.6) |  |  |  |  |  |  |  |  |  |  |
| **Fluoroquinolone** | **R1**  **n/N (%)** | 15/128 (11.7) | 13/128 (10.2) | 116/128 (90.6) |  |  |  |  |  |  |  |  |  |  |  |
|  | **R2**  **n/N (%)** | 15/17 (88.2) | 13/15  (86.7) | 116/139 (83.5) |  |  |  |  |  |  |  |  |  |  |  |
|  | **P-value** | 0.249 | 0.3665 | <0.005 |  |  |  |  |  |  |  |  |  |  |  |
|  | **Odds ratio**  **(95% CI)** | 2.59  (0.56-11.83) | 2.2  (0.48-10.2) | 7.57  (3.21-17.82) |  |  |  |  |  |  |  |  |  |  |  |
| **Trimethoprim-sulphamethoxazole** | **R1** | 16/139 (11.5) | 14/139 (10.1) |  |  |  |  |  |  |  |  |  |  |  |  |
|  | **R2** | 16/17 (94.1) | 14/15  (6.7) |  |  |  |  |  |  |  |  |  |  |  |  |
|  | **P-value** | 0.209 | 0.314 |  |  |  |  |  |  |  |  |  |  |  |  |
|  | **R1%**  **n/N (%)** | 3.77  (0.5-29.6) | 3.25  (0.41-25.7) |  |  |  |  |  |  |  |  |  |  |  |  |
| **Cefoperazone-sulbactam** | **R1**  **n/N (%)** | 14/15 (93.3) |  |  |  |  |  |  |  |  |  |  |  |  |  |
|  | **R2**  **n/N (%)** | 14/17 (82.4) |  |  |  |  |  |  |  |  |  |  |  |  |  |
|  | **P-value** | <0.005 |  |  |  |  |  |  |  |  |  |  |  |  |  |
|  | **Odds ratio**  **(95% CI)** | 704.67  (68.7-7231) |  |  |  |  |  |  |  |  |  |  |  |  |  |

R1 is the number of isolates resistant to both row and column antimicrobial / number of isolates resistant to row antimicrobial (%) whereas R2 is the number of isolates resistant to both row and column antimicrobial / number of isolates resistant to column antimicrobial. P-value for difference was calculated using Chi-square test. Odds-ratio was calculated using binary logistic regression and is listed with 95% confidence interval (95% CI). Two-sided p-value has been reported. n: number of isolates resistant to both row and column antimicrobial; N (in R1): number of isolates resistant to row antimicrobial; and N (in R2): number of isolates resistant to column antimicrobial
