# Supplementary material for: Trends in antimicrobial resistance amongst pathogens isolated from blood and cerebrospinal fluid cultures in Pakistan (2011-2015): A retrospective cross-sectional study
Source: PLoS One. 2021 Apr 26;16(4):e0250226. doi: 10.1371/journal.pone.0250226 (PMC8075205; doi:10.1371/journal.pone.0250226)
Supplement: S3 Table — (DOCX) [file pone.0250226.s003.docx]

**S3 Table. Co-resistance patterns in *Acinetobacter***

| **Antimicrobial** | **Variables** | **Trimethoprim-Sulphamethoxazole** | **Fluoroquinolone** | **Doxycycline** | **Tobramycin** | **Gentamicin** | **Amikacin** | **Piperacillin-tazobactam** | **Cefoperazone-Sulbactam** | **Cephalosporin** | **Penicillin & β-lactamase Inhibitors** |
| --- | --- | --- | --- | --- | --- | --- | --- | --- | --- | --- | --- |
| **Carbapenem** | **R1%**  **n/N (%)** | 96/ 104  (92.3) | 98/ 104  (94.2) | 54/ 104  (51.9) | 78/ 104  (75) | 101/ 104 (97.1) | 98/ 104  (94.2) | 103/ 104  (99) | 54/ 104  (51.9) | 103/ 104  (99) | 104/ 104  (100) |
|  | **R2%**  **n/N (%)** | 96/ 96  (100) | 98/ 99  (99) | 54/ 55  (98.2) | 78/ 79  (98.7) | 101/ 103 (98.1) | 98/ 100  (98) | 103/ 104  (99) | 54/ 54  (100) | 103/ 106 (97.2) | 104/ 108  (96.3) |
|  | **P-value** | <0.005 | <0.005 | 0.3587 | 0.0583 | 0.01 | 0.0269 | <0.005 | 0.118 | 0.073 | NA |
|  | **Odds ratio**  **(95% CI)** | NA | 49  (4.41-544.8) | 3.24  (0.33-32.18) | 9  (0.9-90.33) | 33.67  (3.47-326.38) | 16.33  (1.95-136.93) | 309  (15.38-6207.15) | NA | 34.33  (1.71-689.68) | NA |
| **Penicillin & β-lactamase Inhibitors** | **R1%**  **n/N (%)** | 96/ 108  (88.9) | 99/ 108  (91.7) | 55/ 108  (50.9) | 79/108  (73.1) | 103/ 108 (95.4) | 100/ 108 (92.6) | 104/ 108 (96.3) | 54/ 108  (50) | 106/ 108 (98.1) |  |
|  | **R2%**  **n/N (%)** | 96/ 96  (100) | 99/ 99  (100) | 55/ 55  (100) | 79/ 79  (100) | 103/ 103 (100) | 100/ 100 (100) | 104/ 104 (100) | 54/ 54  (100) | 106/ 106 (100) |  |
|  | **P-value** | NA | NA | NA | NA | NA | NA | NA | NA | NA |  |
|  | **Odds ratio**  **(95% CI)** | NA | NA | NA | NA | NA | NA | NA | NA | NA |  |
| **Cephalosporin** | **R1%**  **n/N (%)** | 96/ 106  (90.6) | 99/ 106  (93.4) | 55/ 106  (51.9) | 79/ 106  (74.5) | 103/ 106 (97.2) | 99/ 106  (93.4) | 103/ 106 (97.2) | 54/ 106  (50.9) |  |  |
|  | **R2%**  **n/N (%)** | 96/ 96  (100) | 99/ 99  (100) | 55/ 55  (100) | 79/ 79  (100) | 103/ 103 (100) | 99/ 100  (99) | 103/ 104  (99) | 54/ 54  (100) |  |  |
|  | **P-value** | 0.0114 | 0.0062 | 0.2385 | 0.0703 | <0.005 | 0.1433 | 0.073 | 0.4953 |  |  |
|  | **Odds ratio**  **(95% CI)** | NA | NA | NA | NA | NA | 14.14  (0.8-250.9) | 34.33  (1.71-689.68) | NA |  |  |
| **Cefoperazone-sulbactam** | **R1%**  **n/N (%)** | 48/ 54  (88.9) | 53/ 54  (98.1) | 29/ 54  (53.7) | 42/ 54  (77.8) | 53/ 54  (98.1) | 52/ 54  (96.3) | 54/ 54  (100) |  |  |  |
|  | **R2%**  **n/N (%)** | 48/ 96  (50) | 53/ 99  (53.5) | 29/ 55  (52.7) | 42/ 79  (53.2) | 53/ 103  (51.5) | 52/ 100  (52) | 54/ 104  (51.9) |  |  |  |
|  | **P-value** | 1 | 0.0314 | 0.5636 | 0.2777 | 0.3632 | 0.2702 | 0.118 |  |  |  |
|  | **Odds ratio**  **(95% CI)** | 1  (0.3-3.32) | 9.22  (1.11-76.49) | 1.25  (0.59-2.66) | 1.61  (0.68-3.8) | 4.24  (0.46-39.24) | 3.25  (0.63-16.88) | NA |  |  |  |
| **Piperacillin-tazobactam** | **R1%**  **n/N (%)** | 96/ 104  (92.3) | 98/ 104  (94.2) | 54/ 104  (51.9) | 78/ 104  (75) | 102/ 104 (98.1) | 99/ 104  (95.2) |  |  |  |  |
|  | **R2%**  **n/N (%)** | 96/ 96  (100) | 98/ 99  (99) | 54/ 55  (98.2) | 78/ 79  (98.7) | 102/ 103  (99) | 99/ 100  (99) |  |  |  |  |
|  | **P-value** | <0.005 | 0.0016 | 0.3587 | 0.0583 | <0.005 | <0.005 |  |  |  |  |
|  | **Odds ratio**  **(95% CI)** | NA | 49  (4.41-544.8) | 3.24  (0.33-32.18) | 9  (0.9-90.33) | 153  (10.69-2189.28) | 59.4  (5.2-678.07) |  |  |  |  |
| **Amikacin** | **R1%**  **n/N (%)** | 91/ 100  (91) | 94/ 100  (94) | 54/ 100  (54) | 76/ 100  (76) | 98/ 100  (98) |  |  |  |  |  |
|  | **R2%**  **n/N (%)** | 91/ 96  (94.8) | 94/ 99  (94.9) | 54/ 55  (98.2) | 76/ 79  (96.2) | 98/ 103  (95.1) |  |  |  |  |  |
|  | **P-value** | 0.0432 | 0.0185 | 0.0301 | 0.0312 | <0.005 |  |  |  |  |  |
|  | **Odds ratio**  **(95% CI)** | 6.07  (1.24-29.65) | 9.4  (1.8-49.06) | 8.22  (0.97-69.28) | 5.28  (1.17-23.73) | 29.4  (3.97-217.71) |  |  |  |  |  |
| **Gentamicin** | **R1%**  **n/N (%)** | 95/ 103  (92.2) | 98/ 103  (95.1) | 55/ 103  (53.4) | 78/ 103  (75.7) |  |  |  |  |  |  |
|  | **R2%**  **n/N (%)** | 95/ 96  (99) | 98/ 99  (99) | 55/ 55  (100) | 78/ 79  (98.7) |  |  |  |  |  |  |
|  | **P-value** | <0.005 | <0.005 | 0.0257 | 0.0179 |  |  |  |  |  |  |
|  | **Odds ratio**  **(95% CI)** | 47.5  (4.73-477.12) | 78.4  (7.34-837.33) | NA | 12.48  (1.33-116.89) |  |  |  |  |  |  |
| **Tobramycin** | **R1%**  **n/N (%)** | 72/ 79  (91.1) | 76/ 79  (96.2) | 50/ 79  (63.3) |  |  |  |  |  |  |  |
|  | **R2%**  **n/N (%)** | 72/ 96  (75) | 76/ 99  (76.8) | 50/ 55  (90.9) |  |  |  |  |  |  |  |
|  | **P-value** | 0.2984 | 0.0109 | <0.005 |  |  |  |  |  |  |  |
|  | **Odds ratio**  **(95% CI)** | 2.14  (0.62-7.38) | 6.61  (1.53-28.52) | 8.28  (2.85-24.05) |  |  |  |  |  |  |  |
| **Doxycycline** | **R1%** | 51/ 55  (92.7) | 55/ 55 (100) |  |  |  |  |  |  |  |  |
|  | **R2%** | 51/ 96  (53.1) | 55/ 99  (55.6) |  |  |  |  |  |  |  |  |
|  | **P-value** | 0.196 | <0.005 |  |  |  |  |  |  |  |  |
|  | **R1%**  **n/N (%)** | 2.27  (0.64-8.03) | NA |  |  |  |  |  |  |  |  |
| **Fluoroquinolone** | **R1%**  **n/N (%)** | 93/ 99  (93.9) |  |  |  |  |  |  |  |  |  |
|  | **R2%**  **n/N (%)** | 93/ 96  (96.9) |  |  |  |  |  |  |  |  |  |
|  | **P-value** | <0.005 |  |  |  |  |  |  |  |  |  |
|  | **Odds ratio**  **(95% CI)** | 31  (6.18-155.58) |  |  |  |  |  |  |  |  |  |

R1 is the number of isolates resistant to both row and column antimicrobial / number of isolates resistant to row antimicrobial (%) whereas R2 is the number of isolates resistant to both row and column antimicrobial / number of isolates resistant to column antimicrobial. P-value for difference was calculated using Chi-square test. Odds-ratio was calculated using binary logistic regression and is listed with 95% confidence interval (95% CI). Two-sided p-value has been reported. n: number of isolates resistant to both row and column antimicrobial; N (in R1): number of isolates resistant to row antimicrobial; and N (in R2): number of isolates resistant to column antimicrobial.
