# Supplementary material for: Trends in antimicrobial resistance amongst pathogens isolated from blood and cerebrospinal fluid cultures in Pakistan (2011-2015): A retrospective cross-sectional study
Source: PLoS One. 2021 Apr 26;16(4):e0250226. doi: 10.1371/journal.pone.0250226 (PMC8075205; doi:10.1371/journal.pone.0250226)
Supplement: S4 Table — (DOCX) [file pone.0250226.s004.docx]

**S4 Table. Co-resistance patterns in *Salmonella enterica* serovar Typhi**

| **Antimicrobial** | **Variables** | **Trimethoprim-sulphamethoxazole** | **Fluoroquinolone** | **Nalidixic acid** |
| --- | --- | --- | --- | --- |
| **Penicillin** | **R1**  **n/N (%)** | 4/5  (80) | 4/5  (80) | 4/5  (80) |
|  | **R2**  **n/N (%)** | 4/49  (8.2) | 4/86  (4.7) | 4/ 100  (4) |
|  | **P-value** | 0.172 | 1 | 0.356 |
|  | **Odds ratio**  **(95% CI)** | 5.24  (0.567-48.55) | 1.073  (0.114-10.93) | 0.333  (.033-3.348) |
| **Nalidixic acid** | **R1**  **n/N (%)** | 46/100  (46) | 86/100  (86) |  |
|  | **R2**  **n/N (%)** | 46/49  (93.9) | 86/86  (100) |  |
|  | **P-value** | 0.51 | <0.005 |  |
|  | **Odds ratio**  **(95% CI)** | 1.704  (0.403-7.195) | NA |  |
| **Fluoroquinolone** | **R1**  **n/N (%)** | 39/86  (45.3) |  |  |
|  | **R2**  **n/N (%)** | 39/49  (79.6) |  |  |
|  | **P-value** | 1 |  |  |
|  | **Odds ratio**  **(95% CI)** | 1.079  (.427-2.727) |  |  |

R1 is the number of isolates resistant to both row and column antimicrobial / number of isolates resistant to row antimicrobial (%) whereas R2 is the number of isolates resistant to both row and column antimicrobial / number of isolates resistant to column antimicrobial. P-value for difference was calculated using Chi-square test. Odds-ratio was calculated using binary logistic regression and is listed with 95% confidence interval (95% CI). Two-sided p-value has been reported. n: number of isolates resistant to both row and column antimicrobial; N (in R1): number of isolates resistant to row antimicrobial; and N (in R2): number of isolates resistant to column antimicrobial.
