# Supplementary material for: Trends in antimicrobial resistance amongst pathogens isolated from blood and cerebrospinal fluid cultures in Pakistan (2011-2015): A retrospective cross-sectional study
Source: PLoS One. 2021 Apr 26;16(4):e0250226. doi: 10.1371/journal.pone.0250226 (PMC8075205; doi:10.1371/journal.pone.0250226)
Supplement: S5 Table — (DOCX) [file pone.0250226.s005.docx]

**S5 Table. Co-resistance patterns in *Staphylococcus aureus***

| **Antimicrobial** | **Variables** | **Fluoroquinolone** | **Doxycycline** | **Clindamycin** | **Macrolide** | **Tobramycin** | **Gentamicin** | **Amikacin** | **Trimethoprim-sulphamethoxazole** | **β-lactam** |
| --- | --- | --- | --- | --- | --- | --- | --- | --- | --- | --- |
| **Penicillin** | **R1**  **n/N (%)** | 47/ 95  (49.5) | 24/ 95  (25.3) | 31/ 95  (32.6) | 48/ 95  (50.5) | 58/ 95  (61.1) | 37/ 95  (38.9) | 16/ 95  (16.8) | 43/ 95  (45.3) | 44/ 95  (46.3) |
|  | **R2**  **n/N (%)** | 47/ 47  (100) | 24/ 24  (100) | 31/ 31  (100) | 48/ 48  (100) | 58/ 59  (98.3) | 37/ 37  (100) | 16/ 16  (100) | 43/ 44  (97.7) | 44/ 44  (100) |
|  | **P-value** | 0.2435 | 0.5738 | 0.5494 | 0.2426 | 0.5613 | 0.2877 | 1 | 1 | 0.2502 |
|  | **Odds ratio**  **(95% CI)** | NA | NA | NA | NA | 3.14  (0.27-35.81) | NA | NA | 1.65  (0.14-18.87) | NA |
| **β-lactam** | **R1**  **n/N (%)** | 38/ 44  (86.4) | 13/ 44  (29.5) | 23/ 44  (52.3) | 35/ 44  (79.5) | 33/ 44  (75) | 30/ 44  (68.2) | 16/ 44  (36.4) | 25/ 44  (56.8) |  |
|  | **R2**  **n/N (%)** | 38/ 47  (80.9) | 13/ 24  (54.2) | 23/ 31  (74.2) | 35/ 48  (72.9) | 33/ 59  (55.9) | 30/ 37  (81.1) | 16/ 16  (100) | 25/ 44  (56.8) |  |
|  | **P-value** | <0.005 | 0.2935 | <0.005 | <0.005 | 0.0069 | <0.005 | <0.005 | 0.0322 |  |
|  | **Odds ratio**  **(95% CI)** | 31.67  (10.34-97.02) | 1.64  (0.65-4.14) | 6.3  (2.42-16.38) | 12.26  (4.69-32.1) | 3.23  (1.36-7.68) | 14.39  (5.21-39.75) | NA | 2.42  (1.07-5.49) |  |
| **Trimethoprim-sulphamethoxazole** | **R1**  **n/N (%)** | 27/ 44  (61.4) | 16/ 44  (36.4) | 19/ 44  (43.2) | 25/ 44  (56.8) | 32/ 44  (72.7) | 22/ 44  (50) | 8/ 44  (18.2) |  |  |
|  | **R2**  **n/N (%)** | 27/ 47  (57.4) | 16/ 24  (66.7) | 19/ 31  (61.3) | 25/ 48  (52.1) | 32/ 59  (54.2) | 22/ 37  (59.5) | 8/ 16  (50) |  |  |
|  | **P-value** | 0.0165 | 0.0136 | 0.0265 | 0.1612 | 0.0222 | 0.024 | 0.6538 |  |  |
|  | **Odds ratio**  **(95% CI)** | 2.7  (1.19-6.13) | 3.29  (1.25-8.67) | 2.66  (1.11-6.39) | 1.77  (0.79-3.96) | 2.67  (1.14-6.25) | 2.6  (1.12-6.02) | 1.28  (0.44-3.74) |  |  |
| **Amikacin** | **R1**  **n/N (%)** | 16/ 16  (100) | 7/ 16  (43.8) | 13/ 16  (81.3) | 13/ 16  (81.3) | 16/ 16  (100) | 16/ 16  (100) |  |  |  |
|  | **R2**  **n/N (%)** | 16/ 47  (34) | 7/ 24  (29.2) | 13/ 31  (41.9) | 13/ 48  (27.1) | 16/ 59  (27.1) | 16/ 37  (43.2) |  |  |  |
|  | **P-value** | <0.005 | 0.0502 | <0.005 | <0.005 | <0.005 | <0.005 |  |  |  |
|  | **Odds ratio**  **(95% CI)** | NA | 2.97  (0.97-9.14) | 15.41  (3.95-60.03) | 5.82  (1.54-21.99) | NA | NA |  |  |  |
| **Gentamicin** | **R1**  **n/N (%)** | 32/ 37  (86.5) | 11/ 37  (29.7) | 21/ 37  (56.8) | 29/ 37  (78.4) | 37/ 37  (100) |  |  |  |  |
|  | **R2**  **n/N (%)** | 32/ 47  (68.1) | 11/ 24  (45.8) | 21/ 31  (67.7) | 29/ 48  (60.4) | 37/ 59  (62.7) |  |  |  |  |
|  | **P-value** | <0.005 | 0.3475 | <0.005 | <0.005 | <0.005 |  |  |  |  |
|  | **Odds ratio**  **(95% CI)** | 19.63  (6.48-59.44) | 1.56  (0.61-3.98) | 6.69  (2.62-17.13) | 8.01  (3.09-20.76) | NA |  |  |  |  |
| **Tobramycin** | **R1**  **n/N (%)** | 34/ 59  (57.6) | 19/ 59  (32.2) | 26/ 59  (44.1) | 37/ 59  (62.7) |  |  |  |  |  |
|  | **R2**  **n/N (%)** | 34/ 47  (72.3) | 19/ 24  (79.2) | 26/ 31  (83.9) | 37/ 48  (77.1) |  |  |  |  |  |
|  | **P-value** | 0.0185 | 0.029 | <0.005 | <0.005 |  |  |  |  |  |
|  | **Odds ratio**  **(95% CI)** | 2.72  (1.17-6.32) | 3.23  (1.09-9.57) | 5.36  (1.84-15.62) | 4.28  (1.79-10.27) |  |  |  |  |  |
| **Macrolide** | **R1**  **n/N (%)** | 35/ 48  (72.9) | 15/ 48  (31.3) | 30/ 48  (62.5) |  |  |  |  |  |  |
|  | **R2**  **n/N (%)** | 35/ 47  (74.5) | 15/ 24  (62.5) | 30/ 31  (96.8) |  |  |  |  |  |  |
|  | **P-value** | <0.005 | 0.1273 | <0.005 |  |  |  |  |  |  |
|  | **Odds ratio**  **(95% CI)** | 8.53  (3.43-21.16) | 2.07  (0.8-5.33) | 81.67  (10.4-643.5) |  |  |  |  |  |  |
| **Clindamycin** | **R1**  **n/N (%)** | 24/ 31  (77.4) | 11/ 31  (35.5) |  |  |  |  |  |  |  |
|  | **R2**  **n/N (%)** | 24/ 47  (51.1) | 11/ 24  (45.8) |  |  |  |  |  |  |  |
|  | **P-value** | <0.005 | 0.0852 |  |  |  |  |  |  |  |
|  | **Odds ratio**  **(95% CI)** | 6.56  (2.46-17.5) | 2.28  (0.88-5.92) |  |  |  |  |  |  |  |
| **Doxycycline** | **R1** | 12/ 24  (50) |  |  |  |  |  |  |  |  |
|  | **R2** | 12/ 47  (25.5) |  |  |  |  |  |  |  |  |
|  | **P-value** | 0.8179 |  |  |  |  |  |  |  |  |
|  | **R1%**  **n/N (%)** | 1.11  (0.44-2.8) |  |  |  |  |  |  |  |  |

R1 is the number of isolates resistant to both row and column antimicrobial / number of isolates resistant to row antimicrobial (%) whereas R2 is the number of isolates resistant to both row and column antimicrobial / number of isolates resistant to column antimicrobial. P-value for difference was calculated using Chi-square test. Odds-ratio was calculated using binary logistic regression and is listed with 95% confidence interval (95% CI). Two-sided p-value has been reported. n: number of isolates resistant to both row and column antimicrobial; N (in R1): number of isolates resistant to row antimicrobial; and N (in R2): number of isolates resistant to column antimicrobial.
